# Deciphering intratumoral microbiota in digestive system tumors: mechanisms and emerging therapeutic strategies

**DOI:** 10.3389/fcimb.2026.1829524

**Published:** 2026-06-10

**Authors:** Huamin Zeng, Hanjing Liu, Fan Yang, Jie Li

**Affiliations:** 1 Cancer Prevention and Treatment Institute of Chengdu, Department of Pathology, Chengdu Fifth People’s Hospital (The Second Clinical Medical College, Affiliated Fifth People’s Hospital of Chengdu University of Traditional Chinese Medicine), Chengdu, Sichuan, China; 2 Institute of Herbgenomics, Chengdu University of Traditional Chinese Medicine, Chengdu, Sichuan, China; 3 College of Medical Technology, Chengdu University of Traditional Chinese Medicine, Chengdu, Sichuan, China

**Keywords:** digestive system tumors, intratumoral microbiota, oncogenic mechanisms, prognosis and diagnosis, traditional Chinese medicine strategies, tumor therapy

## Abstract

Digestive system malignant tumors (DSMTs)—including gastric, pancreatic, colorectal, liver, and esophageal cancers—are major contributors to cancer-related deaths worldwide, owing to their increasing incidence and poor prognoses. Improving patient survival hinges on a deeper understanding of the mechanisms driving DSMT initiation, progression, and therapeutic resistance. This review offers a novel perspective by exploring DSMTs through the lens of the emerging concept of intratumoral microbiota. We outline the historical context and diverse origins of intratumoral microbiota, detailing their multifaceted roles in DSMTs pathogenesis. Additionally, we highlight innovative traditional Chinese medicine strategies that modulate the intratumoral microecosystem for DSMTs prevention and therapy. Finally, we critically assess the translational potential and current challenges associated with microbiota-targeted anticancer therapeutics. Adopting a microbiota-centric paradigm opens new avenues for next-generation precision oncology, aiming to improve outcomes for patients with DSMTs.

## 1 Introduction

Malignant tumors of the digestive system (DSMTs), encompassing gastric (GC), colorectal (CRC), pancreatic (PC), liver (LC), and esophageal (EC), remain a leading global health burden, accounting for nearly half of all cancer-related deaths worldwide (Global Cancer Statistics, 2025) (Dee et al., 2025). Despite advances in surgery, chemotherapy, and immunotherapy, DSMTs are frequently diagnosed at advanced stages, characterized by high rates of therapeutic resistance, metastasis, and recurrence, resulting in low 5-year survival rates (Salmond and Fineran, 2015). Thus, uncovering novel pathogenic mechanisms and developing precision-targeted strategies are urgently needed to improve patient outcomes.

The tumor microenvironment (TME) is a key regulator of cancer progression, and intratumoral microbiota (microbial communities within tumor tissues) are an integral yet understudied component of this ecosystem (Nejman and Livyatan, 2020; Galeano Niño et al., 2022; Chang et al., 2025). Advances in high-throughput sequencing technologies have facilitated the precise identification and functional profiling of intratumoral microbiota across more than 30 cancer types, including DSMTs (Cao et al., 2024). Intratumoral microbiota play dual roles in tumor biology: pathogenic strains drive tumor initiation and progression via DNA damage, inflammatory cascade activation (Liu et al., 2023; Zhang et al., 2024), and immunosuppression, while beneficial strains enhance anti-tumor immunity and sensitize tumors to therapy (Fong et al., 2023; Qu et al., 2023). These findings have profoundly altered our understanding of DSMTs pathogenesis, shifting the paradigm from a tumor-cell-centric perspective to one that integrates the microbiota.

Despite burgeoning research interest, significant gaps persist within the field. Firstly, the heterogeneity of intratumoral microbiota across various DSMT subtypes, along with their associations with tumor stages, clinical characteristics, and patient prognosis, remains incompletely characterized (Zhang et al., 2024). Second, while Western medicine has explored various approaches to target intratumoral microbiota, the distinct potential of Traditional Chinese Medicine (TCM)—a holistic medical system with a long history of regulating host-microbe balance—to modulate intratumoral microbiota homeostasis has been largely overlooked in existing reviews (Han et al., 2024). Third, a systematic framework linking “microbiota characteristics, regulatory mechanisms, clinical diagnosis, therapeutic interventions, and prognostic prediction” is lacking, hindering clinical translation (Liao et al., 2025).

**Figure 1:**
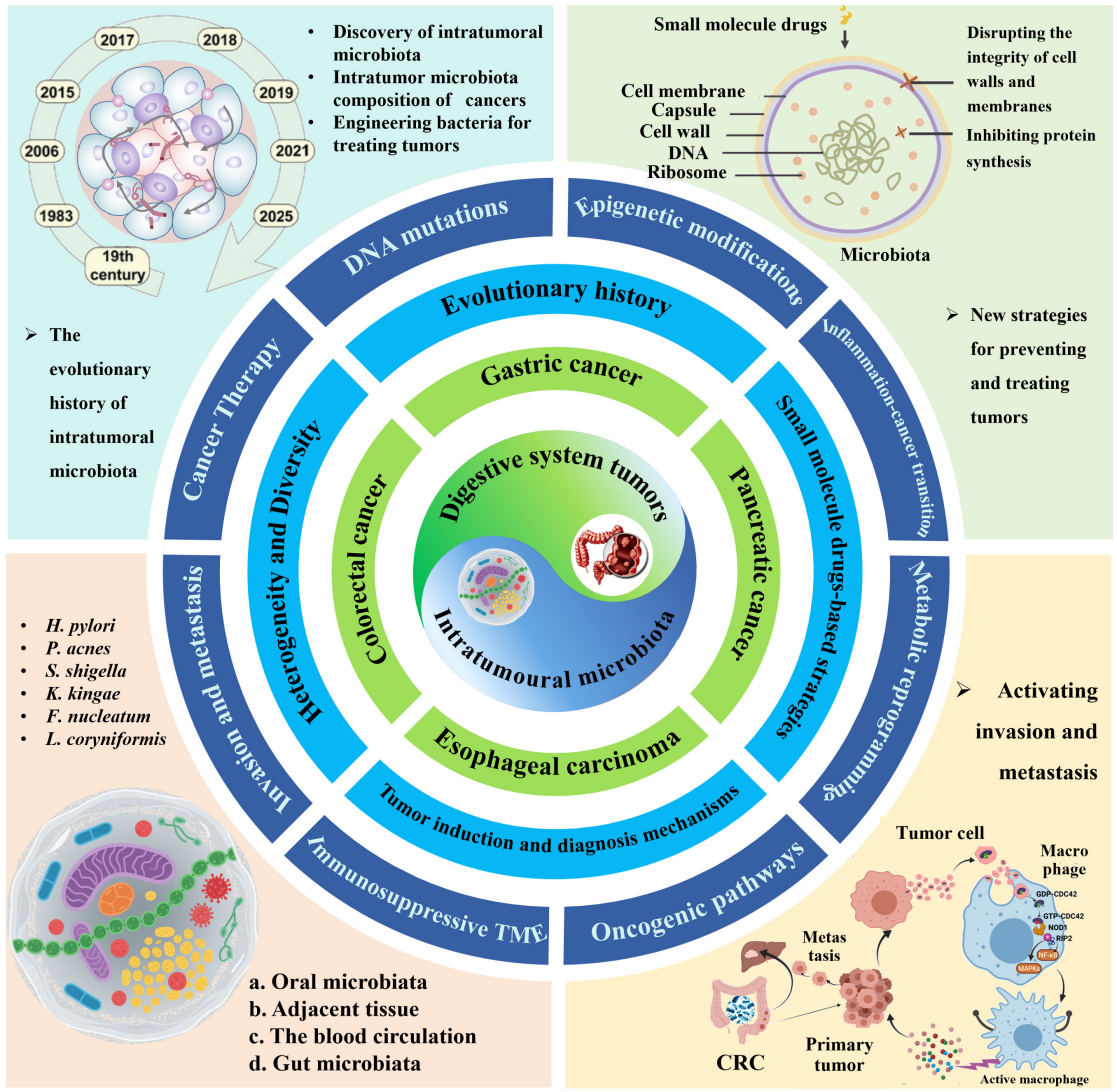
Schematic diagram of this review. The multidimensional landscape of intratumoral microbiota in DSMTs: covering its research evolution, sources, oncogenic mechanisms, and TCM-based therapies targeting this microbiota.

To address these gaps, this review synthesizes recent advancements in intratumoral microbiota research concerning DSMTs (**Figure 1**). We first outline the evolutionary history and diverse origins of intratumoral microbiota, followed by a detailed analysis of their compositional heterogeneity across major DSMT subtypes. Subsequently, we examine the multifaceted mechanisms by which intratumoral microbiota drive tumorigenesis (e.g., DNA damage, inflammation, immunosuppression) and influence therapeutic responses. A central focus is placed on innovative TCM strategies—including compound prescriptions, active monomers, and microbiota modulation—that target intratumoral microbiota for DSMT prevention and treatment, thereby integrating TCM and Western medicine perspectives in this emerging field. Finally, we discuss the clinical potential of intratumoral microbiota as diagnostic and prognostic biomarkers, along with the challenges of translating microbiota-targeted therapies into clinical practice.

## 2 Historical era of intratumoral microbiota

**Figure 2:**
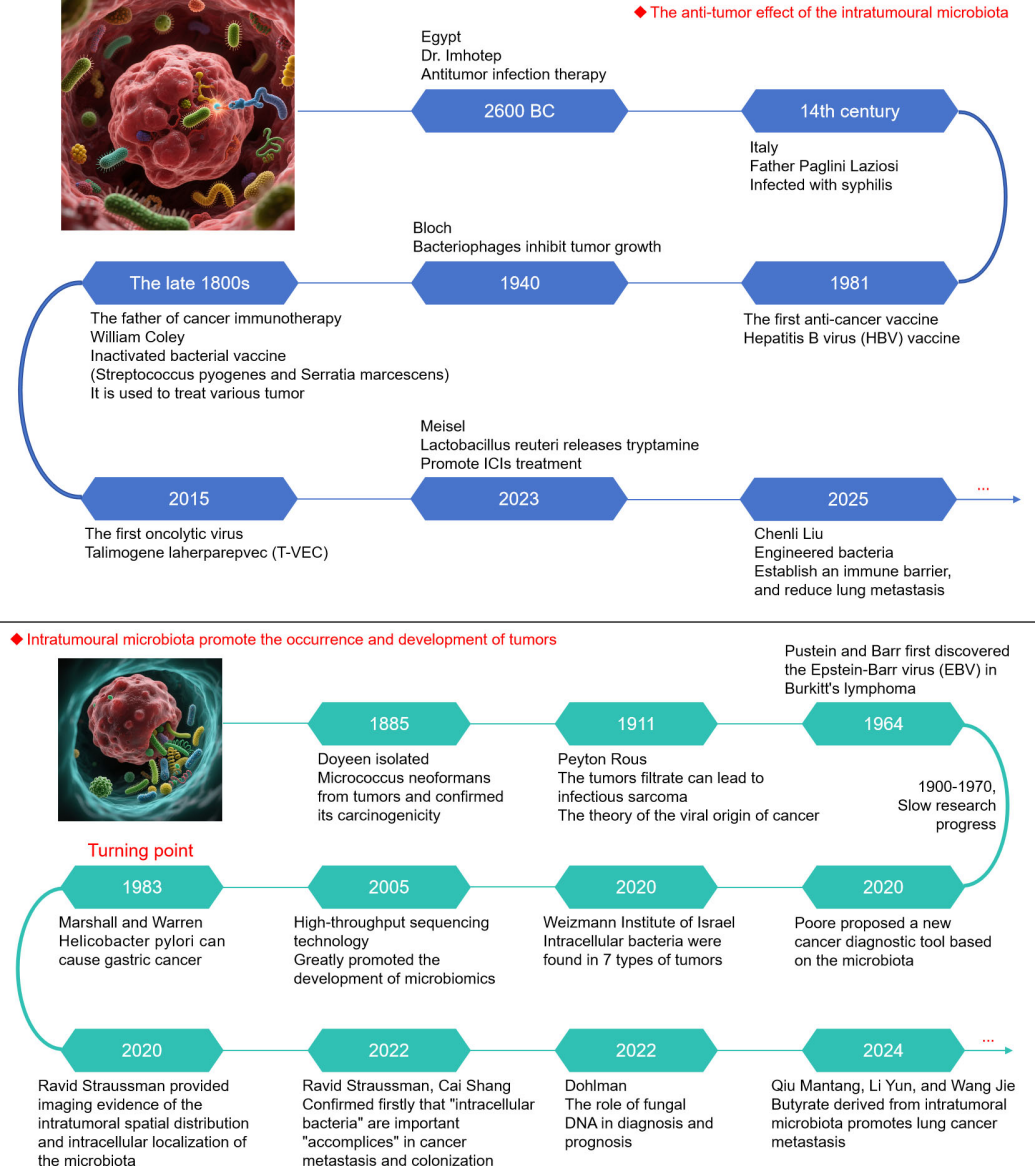
The evolutionary history of intratumoral microbiota, highlighting landmark discoveries, mechanistic insights, and other pivotal advances in the field.

The concept of microbial inhabitants within tumors has evolved in parallel with an expanding understanding of host–microbiota interactions (**Figure 2**). In the 14th century, Pelligrino Laziosi attempted to treat cancer using syphilitic infection. The therapeutic potential of microbes was further explored in 1868, when German physician Friedrich Busch observed spontaneous tumor regression and subsequently infected cancer patients with erysipelas, achieving notable therapeutic effects (Xie et al., 2022). In 1940, Bloch demonstrated that bacteriophages can interact with malignant cells and suppress tumor growth, suggesting viruses as anticancer agents (Cong et al., 2024). The 21st century has seen rapid advances in microbe-centered cancer research. In 2015, the FDA approved talimogene laherparepvec (T-VEC, marketed as Imlygic) for advanced melanoma, heralding a new class of immunotherapeutics (Chesney and Ribas, 2023; Shalhout and Miller, 2023). More recently, Liu et al. (2025) precisely engineered tumor-colonizing bacteria, inducing complete primary tumor regression and establishing durable systemic immunity (Xu et al., 2025).

Nonetheless, microbiota are more often accomplices than adversaries of cancer. In 1885, Doehle isolated Micrococcus neoformans from tumors and demonstrated its oncogenic potential. A major turning point occurred in 1983 when Marshall and Warren isolated *Helicobacter pylori* (*H. pylori*) and demonstrated its causative role in GC,significantly shifting attention to bacteria as carcinogens (Chen et al., 2023; Duan et al., 2025). The advent of high-throughput sequencing in 2005 revolutionized microbiome research by enabling precise taxonomic and functional profiling of intratumoral microbiota (Wang et al., 2021). In 2020, landmark studies by Israeli teams revealed bacteria as integral components of multiple cancer types, leading to the coining of the term “intratumoral microbiota” (Nejman and Livyatan, 2020). Poore et al. analyzed over 30 cancer types and proposed microbiome-based diagnostics (Jiang et al., 2023). In 2022, the same team expanded their research to include fungi, characterizing their intracellular niches and synergistic interactions with bacteria across 35 cancer types (Narunsky-Haziza et al., 2022). In 2024, Qiu, Li, and Wang discovered that intratumoral microbiota trigger LC recurrence. These findings confirm the role of intratumoral microbiota in cancer progression and underscore their prognostic and therapeutic implications (Ma et al., 2024).

## 3 Normal human microbiota

### 3.1 Distribution

The human microbiota are extensively established across various body sites, including the skin, respiratory tract, gastrointestinal tract, and genitourinary tract. Of these, the gastrointestinal tract represents the largest and most significant microbial reservoir, accounting for over 90% of the total microbial biomass. Specific characteristics define the microbiota of different sites: the oral cavity hosts over 700 microbial species; the stomach harbors acid-tolerant microbiota such as *H. pylori*and and *Lactobacillus*; and the intestinal tract, particularly the colon, exhibits the highest microbial density, dominated by phyla including *Firmicutes*, *Bacteroidetes*, *Actinobacteria*, and *Proteobacteria*. In contrast, the small intestine maintains a low microbial load due to rapid peristalsis and bile acid secretion (Gebrayel et al., 2022). Together, these constitute a continuous and diverse distribution of gastrointestinal microbiota from the oral cavity to the intestinal tract.

### 3.2 Physiological functions of gastrointestinal microbiota

Gastrointestinal microbiota are closely symbiotic with the host, playing irreplaceable roles in digestive organ homeostasis and DSMT initiation/progression. Their importance is highlighted by four key roles: (i) they directly impact the physiology of the gastrointestinal tract, liver, and pancreas, regulating digestive enzyme secretion, bile acid metabolism, and energy homeostasis (Wong-Rolle et al., 2021); (ii) they serve as the primary source of intratumoral microbiota in DSMTs: intestinal/oral microbiota can translocate into tumors when the mucosal barrier is impaired, facilitated by mechanisms such as damage, contiguous colonization, or hematogenous dissemination (Hays et al., 2024); (iii) their balance is critical for the prevention of DSMTs—dysbiosis (elevated pathogens, reduced probiotics) promotes tumorigenesis through chronic inflammation, DNA damage, and immune suppression, while *Bifidobacterium* and *Lactobacillus* exert anti-tumor effects via anti-inflammatory and immune-enhancing actions (Fernandez et al., 2025); (iv) they modulate DSMT therapeutic responses. SCFAs enhance immune checkpoint inhibitor efficacy, while dysbiosis-induced intestinal barrier damage increases chemotherapy toxicity (Mukhopadhya and Louis, 2025).

## 4 Intratumoral microbiota in DSMTs

This section discusses candidate reservoirs, the compositional heterogeneity of intratumoral microbiota in digestive system malignant tumors, and its correlation with gut microbiota, to understand oncogenic mechanisms and clinical applications.

### 4.1 Candidate reservoirs

**Figure 3:**
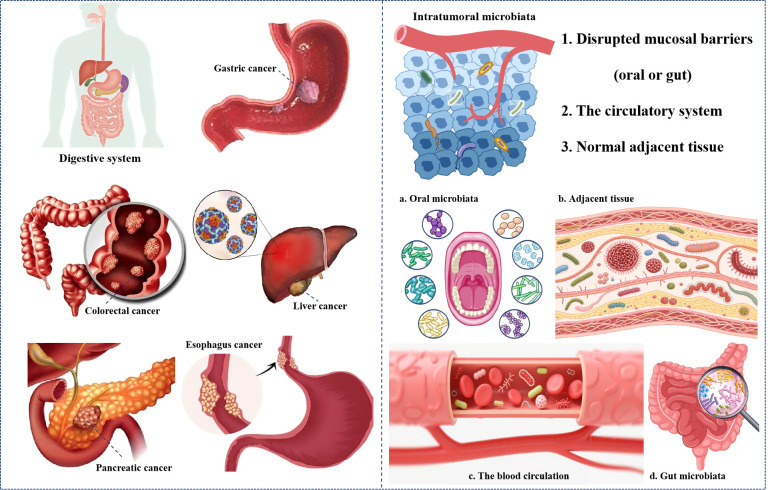
Candidate reservoirs of intratumor microbiota. The oral and gut microbiota could serve as candidate reservoirs of intratumoral microbiota. The TME is prone to promoting the migration of microbiota from normal into cancer sites. For microbiota that enter the bloodstream from various sites, the pressure/concentration gradient inside and outside tumor cells may aid their translocation to the TME. Since a large number of microbiota inhabit mucosal organs (e.g., digestive tract and oral cavity), impairment of the mucosal barriers may facilitate the entry of the intratumoral microbiota.

Elucidating the origins and molecular pathways of intratumoral microbiota is essential for optimizing therapeutic efficacy. Three principal sources have been proposed (**Figure 3**): (i) Translocation across compromised mucosal barriers: The aerodigestive tract is typically lined by epithelial barriers that prevent microbial entry. However, during neoplastic progression, ulceration, or therapy-induced damage, gastrointestinal and oral microbiota can infiltrate tumors (Paone and Cani, 2020). For example, Zhu et al. demonstrated that orally administered *Akkermansia*can traverse compromised mucosal barriers and enter tumor cells (Zhu et al., 2023). (ii) Contiguous colonization from adjacent healthy tissues: Metataxonomic surveys indicate significant compositional overlap between tumors and the surrounding mucosa. This suggests that within the hypoxic and immunosuppressed TME microbes preferentially colonize tumor cells, thereby disrupting the ecological balance between the tumor and its adjacent healthy tissues (Sun et al., 2023; Cai and Zhu, 2024). (iii) Hematogenous dissemination: Oral and intestinal microbiota can disseminate to tumors via compromised vasculature. Specifically, intravenously administered *Fusobacterium nucleatum* (*F. nucleatum*) localizes to colorectal tumors by anchoring to the cancer-associated disaccharide Gal-GalNAc via its surface lectin Fap2 (Jiang et al., 2023).

### 4.2 Intratumoral microbiota heterogeneity across different DSMTs

TMEs vary considerably across cancer types, and the identity and density of intratumoral microbiota also vary (Galeano Niño et al., 2022; Zhang et al., 2024). Intratumoral microbiota composition and quantity vary by tumor type and stage, thereby exerting specific carcinogenic or protective effects. **Supplementary Table 1** presents a systematic overview of intratumoral microbiota in digestive tract tumors. The list comprises the predominant bacteria alongside less abundant, though clinically relevant, fungal and viral components.

#### 4.2.1 Gastric cancer

*H.* pylori and EBV were detected in gastric cancer, and it was found that they are associated with precancerous lesions (Chen et al., 2023; Li et al., 2025). Research indicates significant microbial shifts across disease stages, from gastritis to GC. *H. pylori*, a bacterium considered oncogenic, exhibits higher abundance in early-stage GC compared to non-cancerous tissues, with its levels decreasing as the disease progresses (Yang et al., 2019). Similar trends are observed for *Propionibacterium acnes* (*P. acnes*), TM7, *Porphyromonas*, *Neisseria*, *Streptococcus*, and *Prevotella* (Zeng et al., 2024). Conversely, *Lactobacillus coryniformis* (*L. coryniformis*) and *Lachnospiraceae* increase as GC develops. Peng et al. discovered *Oceanobacterium*, *Methylobacterium*, and *Syntrophomonas* are enriched in tumors, with *Methylobacterium* linked to poor prognosis in GC patients (Peng and Liu, 2022). Genera such as *Moraxella*, *Vibrio*, *Paludibacter*, *Agrobacterium*, *Clostridium*, and the family Comamonadacea*e* have been used to predict GC risk.

#### 4.2.2 Pancreatic cancer

The poor prognosis of PDAC, the most prominent PC subtype, is likely influenced by abundant intratumoral microbiota (Leng et al., 2024). PDAC microbiota differ markedly from those in healthy tissues, particularly *Pseudomonas* and *Kingella kingae* (*K. kingae*), which are present in high levels in tumors (Qian et al., 2023). *H. pylori* also colonizes pancreatic cells, activating molecular pathways that promote tumorigenesis (Jin et al., 2022). Pancreatic *H. pylori* strains differ from duodenal strains, suggesting that *H. pylori* migrating from the duodenum to the pancreas might have undergone mutations, or that the pancreatic strains originated from other sources (Chang et al., 2024). Other intratumoral microbes, including *Acinetobacter*, *Pseudomonas*, *sphingomyelinases*, and *Malassezia*, have been identified in basal-like PDAC tumors, all of which are known to display strong carcinogenic effects (Guo et al., 2021).

#### 4.2.3 Colorectal cancer

*F. nucleatum*, *Anaerococcus vaginalis*, *H. pylori* and enterotoxigenic *B. fragilis* (ETBF) strains are key drivers of CRC development and progression (Zhang et al., 2024; Shi and Ren, 2025). Tumor tissues exhibit increased abundance of *Bacteroides*, *Prevotella*, *Lactococcus*, *Fusobacterium*, and *Streptococcus* compared with normal tissues; in contrast, *Pseudomonas* and *S. shigella* predominate in adjacent healthy tissue (Long et al., 2024). Tito et al. reported *Anaerococcus vaginalis*, *Dialister pneumosintes*, and *Prevotella intermedia* displayed significant differences in healthy, adenoma and carcinoma groups (Tito et al., 2024). Microbial composition also varies by tumor location: *Bifidobacterium* and *Romboutsia* are more prevalent in left-sided CRC, while *Haemophilus* and *Veillonella* are enriched in right-sided CRC (Mao and Huang, 2025). Notably, *H. pylori* is not typically an intratumoral microbe in CRC in clinical settings. As a result, studies on its immune effects in colorectal tumors are primarily derived from experimental models rather than clinical CRC specimens, which reflects the tumor-type specificity of its biological roles.

#### 4.2.4 Liver cancer

Huang et al. demonstrated that the liver harbors microbes, with *γ*-*proteobacteria* being more abundant in tumor tissue than in healthy tissue (Gu et al., 2024). Distinct microbial signatures characterize hepatocellular carcinoma (HCC), varying by its etiology. For instance, cirrhotic HCC shows an increase in *Lactococcus* and *Streptococcaceae*, patterns not observed in non-cirrhotic HCC (Sun et al., 2023; Dzutsev and Trinchieri, 2025). Similarly, Qu et al. reported an increased *Enterobacteriaceae* count in HCC, a finding contrary to observations in combined HCC and intrahepatic cholangiocarcinoma (ICC) cases. They also noted a reduced *Rickettsiaceae* abundance (Wang et al., 2025).

#### 4.2.5 Esophageal cancer

Increased abundances of *F. nucleatum*, *Prevotellaceae*, and *Streptococcus*, along with a decline in *Proteobacteria*, have been associated with reduced survival rates in patients with EC (Wu and Leng, 2023; Wu et al., 2024). Yamamura et al. (2016) observed higher CCL20 levels in *F. nucleatum*-positive tumor sites than in negative tumor sites, indicating its role in the “cytokine–cytokine receptor interaction” pathway in EC (Yamamura et al., 2016). Therefore, they proposed *F. nucleatum* as a prognostic biomarker, suggesting that antimicrobial strategies could improve outcomes after esophagectomy. This is further supported by Yamamura et al. (2019), who reported that *F. nucleatum* induces resistance to chemotherapeutics, including docetaxel and 5-fluorouracil, in squamous cell carcinoma cell lines (Yamamura et al., 2019). In a related but distinct finding, enrichment of *Lachnospiraceae* and *Bacteroidaceae* reduces microbial diversity, potentially contributing to a higher incidence of anastomotic leakage.

## 5 Mechanisms of intratumoral microbiota in inducing DSMTs

**Figure 4:**
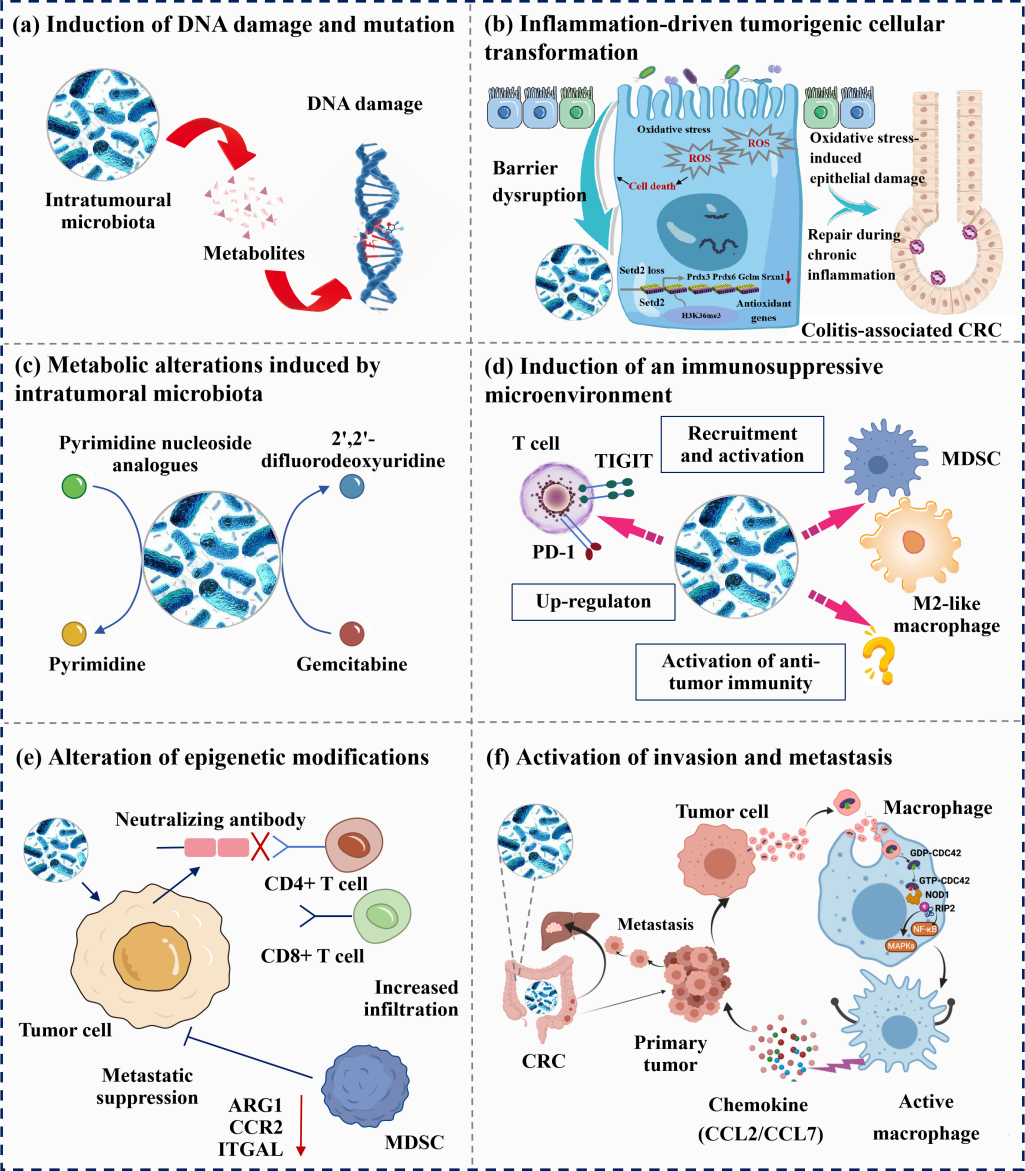
Mechanisms of intratumoral microbiota in inducing DSMTs. **(a)** Induction of DNA damage and mutation, **(b)** Inflammation-driven tumorigenic cellular transformation, **(c)** Metabolic alterations induced by intratumoral microbiota, **(d)** Induction of an immunosuppressive microenvironment, **(e)** Alteration of epigenetic modifications, **(f)** Activation of invasion and metastasis.

Intratumoral microbiota drive the initiation and progression of DSMTs through multiple mechanisms. Among these, carcinogenic microbiota can directly cause DNA damage, induce pro-inflammatory cascades, or trigger other pathways that promote tumorigenesis (Kitano et al., 2025). Furthermore, specific intratumoral microbiota contribute to tumor progression by suppressing the immune system and inhibiting various anti-tumor therapies. A thorough understanding of these mechanisms is therefore crucial for enhancing cancer prognosis and optimizing treatment strategies(**Figure 4**) (Wang et al., 2023).

### 5.1 Induction of DNA damage and mutation

Microbiota can induce genomic mutations leading to cancer. Over 10% of cancers are caused by viruses integrating their genomes into host chromosomes. *Enterobacteriaceae* (e.g., *E. coli*) produce toxins that mutate DNA, contributing to tumor formation (especially CRC) (Dejea et al., 2018; Dziubańska-Kusibab and Berger, 2020; DeStefano Shields et al., 2021). *F. nucleatum* facilitates E-cadherin/β-catenin activation by secreting the adhesin FadA, which upregulates CHEK2 and causes DNA damage in cancer cells (Zhang et al., 2023). *E. coli* expressing EspF and *H. pylori* disrupt DNA repair, causing genomic instability and tumorigenesis. ETBF toxins increase spermine oxidase (SMO) in colonic epithelial cells, provoking SMO-dependent ROS and γ-H2A production, leading to DNA mutations. Inhibiting DNA oxidative damage can reduce microorganism-induced colitis-associated CRC (Xiao et al., 2024). Other pathogenic bacteria that induce genomic mutations include *Campylobacter jejuni* (*C. jejuni*), *Bacteroides fragilis* (*B. fragilis*), and *E. coli.* These bacteria disrupt the DNA of intestinal epithelial cells, thereby contributing to tumorigenesis, through either oxidative or nitrosative DNA damage or the production of cytolethal distending toxin (Jia and Azzi-Martin, 2025).

### 5.2 Alteration of epigenetic modifications

Intratumoral microbiota regulate host epigenetic modifications, including RNA modifications, DNA methylation, histone modifications, and non-coding RNAs, via both direct and indirect mechanisms. These processes are critical for the development of DSMTs.

(i) Direct regulation: Abnormal DNA methylation associated with *H. pylori*, *Kytococcus sidolamus* (*K. sidolamus*), or *Actinomyces oris* (*A. oris*) constitutes a major pathway contributing to gastric adenocarcinoma (Zhou et al., 2024). This can also promote gastric adenocarcinoma metastasis and influence prognosis. Intratumoral *F. nucleatum* abundance correlates with hypermethylation of the CDKN2A promoter CpG island in patients with CRC (Park and Keith, 2024). They also mediate hypermethylation of tumor suppressor gene promoters through the upregulation of DNA methyltransferases in CRC. Another study reported that *F. nucleatum* induces SP1-mediated transcription of the enolase 1-intronic transcript 1, leading to the recruitment of KAT7 histone acetyltransferase, which modifies histone patterns and promotes CRC development (Hong et al., 2021). Moreover, *Mycoplasma hyorhinis* increases m6A levels, causing mitofusin mRNA degradation that enhances mitochondrial fission, thereby facilitating HCC initiation and progression (Qiao et al., 2023).

(ii) Indirect regulation: Microbial metabolites indirectly regulate host epigenetic modifications by acting as cofactors or influencing regulatory enzymes. For example, correlation analysis of intratumoral microbiota and DNA methylation profiles in HCC revealed 10 metabolites closely associated with 25 methylation-associated differentially expressed genes. Short-chain fatty acids also induce epigenetic modifications by affecting histone deacetylase and acetyltransferase activity (Liu et al., 2025). Vitamins B2, B12 and folate also serve as major substrates for methylation. Ma et al. demonstrated that butyrate upregulates H19 expression by promoting M2 macrophage polarization and increasing histone H3 lysine 27 acetylation at the H19 promoter, thereby facilitating LC progression (Ma et al., 2024). Methionine in LC patients is a major methyl donor for protein and DNA methylation, leading to epigenetic reprogramming. ROS from *F. nucleatum* infection initiate MSH2/MSH6-dependent repair, leading to DNA hypermethylation.

### 5.3 Inflammation-driven tumorigenic cellular transformation

Tissue injury resulting from microbial infections recruits neutrophils, triggering inflammation and establishing an inflammatory TME. This TME subsequently evolves into an immunosuppressive environment, facilitating DSMTs progression and diminishing anti-tumor efficiency. Consequently, chronic inflammation is a driver for certain DSMT.

Numerous studies demonstrate that intratumoral microbiota activate pro-inflammatory cascades by stimulating cytokine production via pattern recognition receptors (PRRs) within the TME, thereby promoting tumor progression (Baruch et al., 2021). For example, *F. nucleatum* promotes the proliferation and migration of CRC cells by activating related signaling pathways (Yamane et al., 2022). Kong et al. observed that *F. nucleatum* activates the TLR4 pathway and increases CYP2J2 expression, which catalyzes linoleic acid into 12,13-EpOME, ultimately inducing epithelial–mesenchymal transition (EMT). Furthermore, *F. nucleatum* promotes CRC proliferation and metastasis through mechanisms that include the TLR4/MYD88/NF-κB signaling pathway. For instance, *F. nucleatum* inhibits autophagy in CRC, leading to increased pro-inflammatory cytokines (e.g., IL-8, IL-1β, and TNF-α). Beyond CRC-specific effects, intracellular *Aggregatibacter actinomycetemcomitans* and *F. nucleatum* are known to activate TLR4 in macrophages and downstream NF-κB, stimulating IL-6 secretion in mice. Separately, *P. gingivalis* activates to the proliferation of PC and CRC cells by activating the MAPK signaling pathway via bacterial gingipains (Díaz-Basabe et al., 2024).

Intratumoral microbiota can also induce macropinocytosis, thereby driving inflammation. This process can, for instance, involve Wnt pathway activation, which enhances macrophage proliferation and metabolite uptake. Potential transcriptional targets of Wnt-activated macropinocytosis include RAB5, PDK1, and PAK1 (Zhang et al., 2023). For instance, intratumoral microbiota can increase IL-17 secretion, thereby accelerating tumor growth and progression. Conversely, neutrophil depletion promotes *Akkermansia*in CRC.

### 5.4 Metabolic alterations induced by intratumoral microbiota

**Figure 5:**
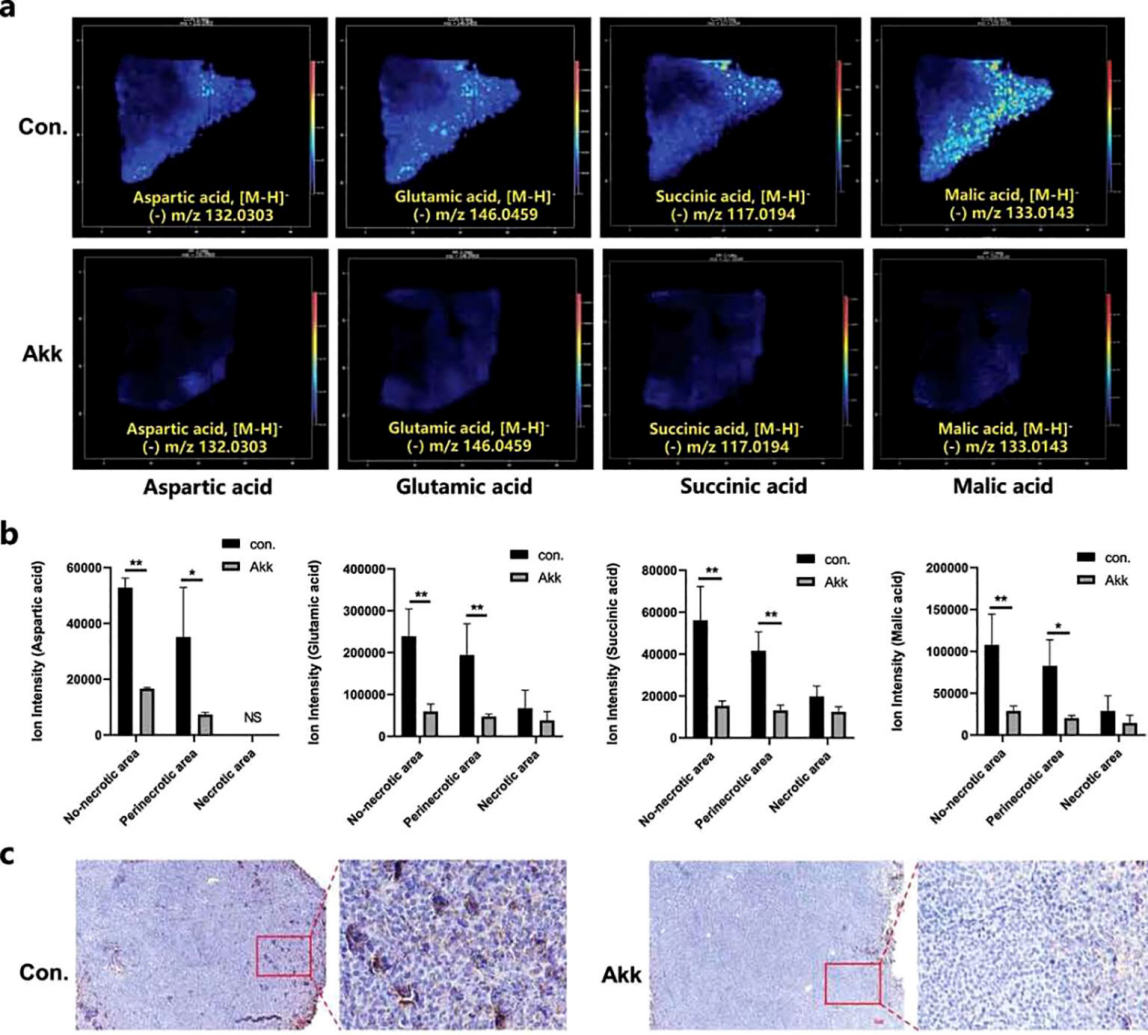
*In situ* visualization of crucial metabolites and metabolic enzymes in the glutamine metabolism pathway. **(a)** Representative MSI images of glutamine metabolism. **(b)** Glu, aspartic acid, succinate, and malic acid levels in the different cancer subregions. **(c)** IHC staining of the metabolic enzymes GLS in Lewis lung cancer tissue section. Copyright (2023) Taylor & Francis Online (Zhu et al., 2023).

The notion that metabolites associated with intratumoral microbiota are intricately involved in cancer progression is increasingly recognized (**Figure 5**). Non-targeted metabolomic analysis of gastric tumors revealed that carbohydrate levels in GC sites—where *H. pylori* and lactic acid bacteria were negatively and positively correlated, respectively—were elevated compared to non-tumor sites. ICC tissues contain abundant *Pseudomonadales*, *Bacillales*, *Clostridiales*, and *Xanthomonadales* (Liu and Jiang, 2025). The number of *Pseudomonas* is elevated in tumor sites and negatively correlated with CA199 levels, suggesting its potential carcinogenic role (Li et al., 2022). Similarly, intratumoral bacterial regions in HCC exhibit substantial enrichment of fatty acids and lipopolysaccharides, both of which have been identified as critical drivers of HCC invasion and metastasis (Filali-Mouncef et al., 2022).

Furthermore, microbiota heterogeneity contributes to variations in metabolic pathways among patients with PDAC, consequently influencing patient survival. Patients with short survival times exhibit higher levels of intratumoral *Actinobacteria* (for example, *Saccharopolyspora* and *Streptomyces*) and *Proteobacteria* (for example, *Pseudoxanthomonas*) (Panebianco et al., 2022). Conversely, patients with prolonged survival harbor intratumoral microbiota primarily composed of of *α-Proteobacteria*, *Flavobacteria*, and *Sphingobacteria*.

### 5.5 Induction of an immunosuppressive microenvironment

**Figure 6:**
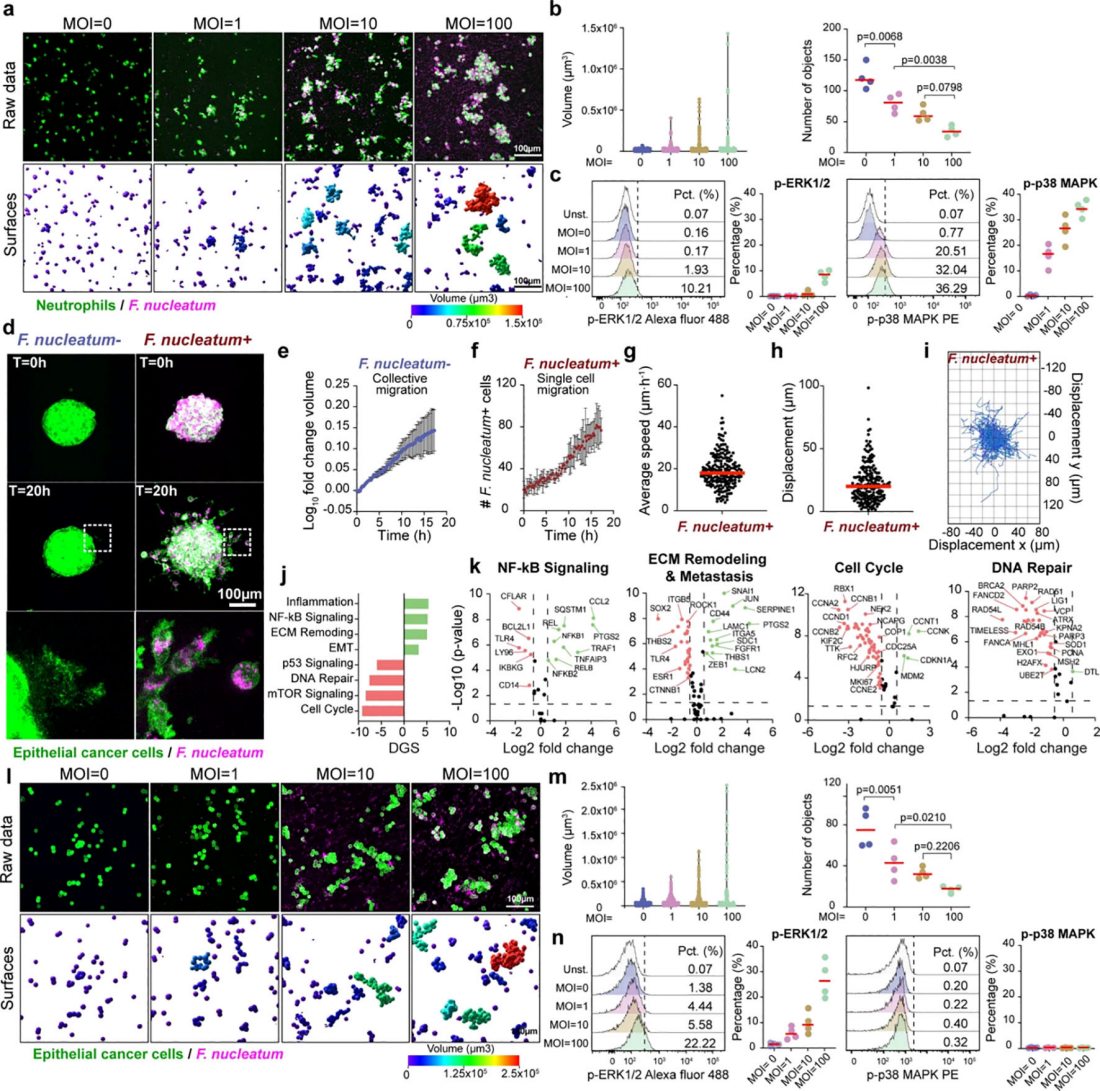
F*. nucleatum* triggers the formation of tumor and immune cells clusters. **(a)** Analysis of cluster formation. **(b)** Violin and dot plots quantifying neutrophil cluster volume and number. **(c)** Flow cytometry of MAPK/ERK phosphorylation. **(d)** Live confocal imaging of invasion. **(e, f)** Volume expansion of uninfected CRC spheroids and number of detached *F. nucleatum*-positive cancer cells (n=3). **(g–i)** Speed, displacement, and trajectories. **(j, k)** Signaling pathway and gene regulation analysis. **(l, m)** Cluster formation and quantification at different MOIs. **(n)** ERK/p38 MAPK phosphorylation levels. Copyright (2022) Nature Portfolio (Galeano Niño et al., 2022).

The immune microenvironment plays a critical role in the growth and metastasis of specific DSMTs. Intratumoral microbiota are implicated in the recruitment of myeloid-derived suppressor cells, thereby inducing an immunosuppressive TME that facilitates immune escape and tumorigenesis (**Figure 6**) (Galeano Niño et al., 2022). For example, *F. nucleatum* inhibits CD8+ T cell infiltration and the secretion of anti-tumor cytokines by recruiting tumor-associated macrophages, establishing an immunosuppressive TME, and increasing PD-L1 expression, thereby increasing the risk of CRC or PC (Jiang et al., 2023). Similarly, regions enriched with *Neisseriaceae* show an increase in M2-like macrophages and a decrease in cytotoxic CD8+ T cells (Juárez Rodríguez et al., 2024). In addition, *Staphylococcus aureus* (*S. aureus*), HBV, and hepatitis C virus promote LC progression by enhancing Treg-mediated immunosuppression (Shehata et al., 2023). Intratumoral microbiota also contribute to immune escape through the inactivation of immune cells. Specifically, *F. nucleatum*-derived Fap2 directly binds to the tyrosine-based inhibitory motif (TIGIT) domain, thereby inhibiting NK and T-cell activity (Pignatelli et al., 2023). Likewise, *H. pylori* inactivates immune cells, increases IL-8 production, and mediates CagA infiltration through interaction with CEACAM1, thereby facilitating GC development (Shrestha et al., 2022). CagA also induces PD-L1 expression in gastric mucosal epithelial cells, which is a recognized marker of GC precancerous lesions.

In addition to bacteria, intratumoral fungi contribute significantly to tumor development. For instance, intratumoral fungi accelerate PDAC progression by activating group 2 innate lymphoid cells (Alam et al., 2022). Therefore, eliminating intratumoral microbiota constitutes an effective strategy to mitigate the immunosuppressive TME or prevent immune cell dysfunction, thereby potentially aiding in the prevention and treatment of CRC, LC or PC.

### 5.6 Activation of invasion and metastasis

Intratumoral microbiota play a critical role in the metastasis of DSMTs, a process that significantly limits the efficacy of anti-tumor therapies. Bacterial enrichment has been identified within metastatic lesions of various DSMTs. Upon entering circulation, intratumoral bacteria harbored by tumor cells induce actin cytoskeleton reorganization, thereby enhancing resistance to fluid shear stress and consequently prolonging tumor cell survival.

EMT is a key driver of invasion and dissemination. Intratumoral microbiota promote EMT via TGFβ/SMAD and PI3K/AKT signaling pathways, leading to enhanced cancer cell metastasis. For example, (i) *F. nucleatum* secretes DNA starvation/stationary-phase protection protein, which drives CRC cell migration through CCL2/CCL7-induced EMT and facilitates *in vivo* metastasis (Yu et al., 2025). (ii) *Candida* downregulates genes involved in cell adhesion, such as PTK2B, CDKN2C, and NET1, thereby contributing to the development of advanced metastatic colon tumors. Additionally, intratumoral microbiota influence EMT and induce the expression of cancer stem cell (CSC) biomarkers. For instance, Bessède et al. demonstrated that *H. pylori-*derived CagA promotes cell migration and invasion *in vitro* by inducing EMT-associated changes and CD44 expression (He et al., 2025). Adhesion between tumor and endothelial cells represents another critical step in metastasis. *F. nucleatum* activates NF-κB signaling through the pattern recognition receptor ALPK1, leading to the upregulation of ICAM1. This consequently promotes the adhesion of endothelial cells to CRC cells and facilitates subsequent metastasis. Furthermore, the intratumoral microbiota establishes a TME conducive to cancer metastasis. Specifically, in CRC, intratumoral *E. coli* disrupts the intestinal vascular barrier via VirF, thereby facilitating bacterial dissemination to the liver and the subsequent recruitment of metastatic cells (Bertocchi et al., 2021).

## 6 Clinical relevance of intratumoral microbiota in DSMTs prevention and treatment

With advances in intratumoral microbiota research, their critical role in the diagnosis, prevention, and treatment of DSMTs is increasingly recognized. The distinct microbial composition within the TME of patients with DSMTs, varying with survival duration, suggests that intratumoral microbial traits can serve as biomarkers (Lu et al., 2024). Patients with long survival durations may also harbor “benign” intratumoral microbiota that exert anti-tumor effects. Consequently, identifying or supplementing these beneficial microbiota could guide DSMTs prevention and treatment (**Supplementary Table 2**).

### 6.1 Diagnosis and prognosis-based applications of intratumoral microbiota

**Figure 7:**
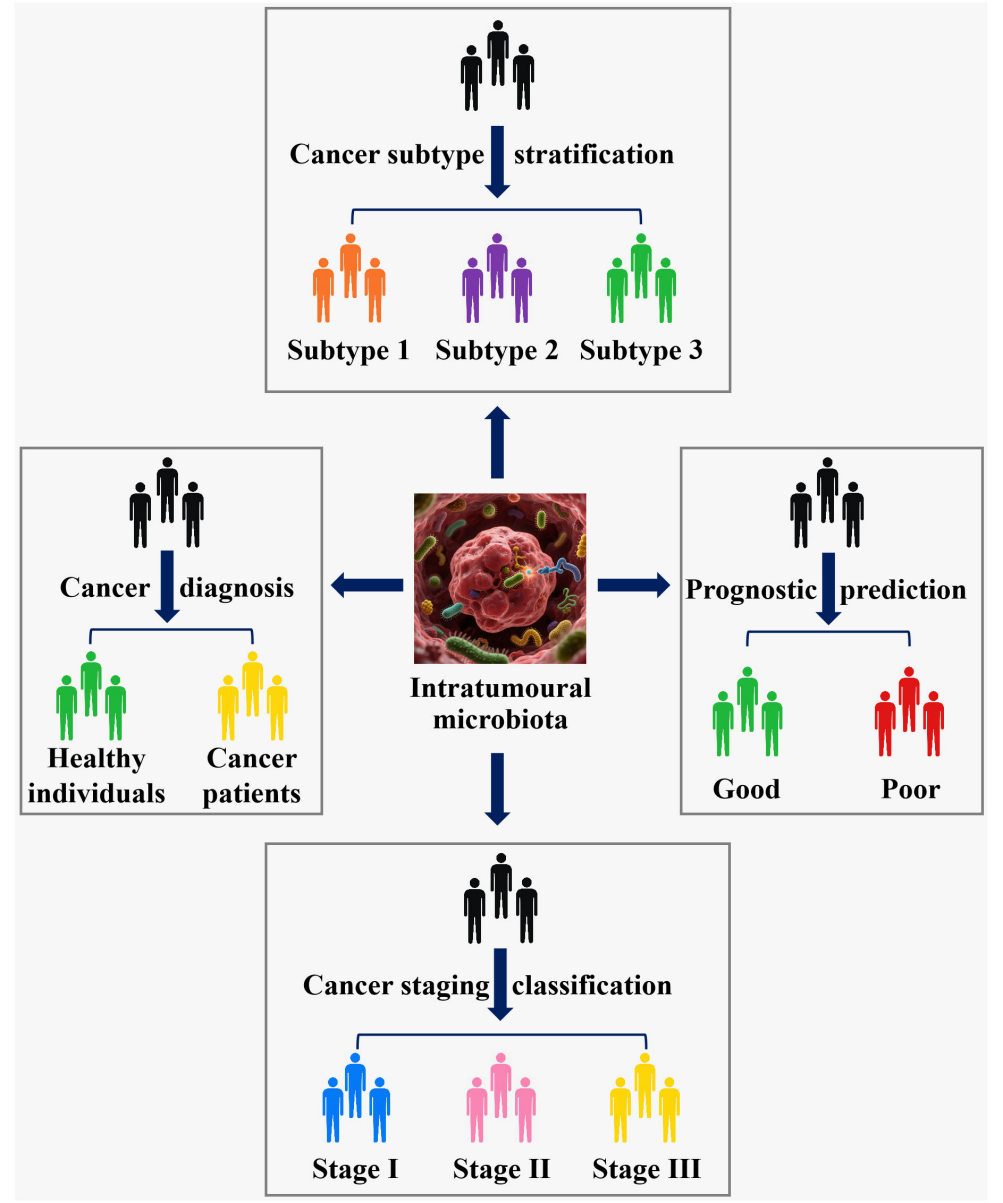
The intratumoral microbiota holds significant potential for clinical applications in oncology. Distinct tumor-associated microbial signatures can effectively distinguish tumor patients from healthy subjects. These diagnostic biomarkers also allow for the differentiation of various cancer subtypes, as well as of the same subtype across different stages. Intratumoral microbiota could serve as a useful tool for cancer diagnosis, prevention, and treatment. The content and heterogeneity of intratumoral microbiota link to clinical outcomes in tumor patients.

The heterogeneity of intratumoral microbiota across different cancer types and healthy tissues suggests their potential as diagnostic and prognostic markers (**Figure 7**). Distinct microbial profiles associated with various DSMTs have been extensively characterized (Jiang et al., 2023; Shi et al., 2024). The intratumoral microbiota of CRC is predominantly composed of *Firmicutes* and *Bacteroidetes*. Conversely, *Proteobacteria* are prevalent in PC, and *Actinobacteria* are predominant in tumors outside the digestive system. Clinical retrospective studies have revealed that *Streptococcus* or *B. fragilis* are associated with an increased risk of CRC, while *F. nucleatum* has been linked to BRAF mutations or microsatellite instability within CRC tissues (Saito and Karasawa, 2025). Intratumoral microbiota can be utilized for diagnosing and identifying cancer subtypes. For example, Mouradov et al. reported that microbial community subtypes within tumors can differentiate patient subgroups across stages I to IV CRC (Mouradov et al., 2023). However, this strategy faces challenges such as low microbiota biomass and susceptibility to false positives from environmental contaminants. Therefore, accurate prediction of tumor formation and progression in clinical practice necessitates a multi-factor approach, integrating blood-based microbiological diagnosis with imaging to reveal the microbiota’s spatial arrangement and intracellular positioning.

Furthermore, intratumoral microbiota are critical determinants of DSMT prognosis, given their established association with patient survival rates. Numerous studies have demonstrated that intratumoral bacterial diversity contributes to improved long-term survival in patients with PDAC (Panebianco et al., 2022). The characteristic composition of intratumoral microbiota, including *Pseudomonas*, *Bacillus clausii*, and *Streptomyces*, is often used to predict survival outcomes in patients with PDAC. Conversely, short survival times in PDAC patients are associated with anaerobic bacteria such as *Proteobacteria*, *Peptoniphilus*, *Bacteroides*, and *Lactobacillus*. Furthermore, a higher abundance of *F. nucleatum* in CRC and EC often indicates poorer survival rates (Qu et al., 2023; Wu et al., 2024). Accordingly, intratumoral microbiota can serve as diagnostic and prognostic markers, guiding prospective therapy for patients in various risk groups.

### 6.2 Cancer therapy based on intratumoral microbiota

Intratumoral microbiota exert bidirectional and context-dependent effects on DSMTs. Pathogenic strains typically promote immunosuppression and tumor progression, whereas beneficial intratumoral microbiota enhance anti-tumor effects or immunity. This duality is strongly influenced by tumor subtype, anatomical site, and experimental models, necessitating clear differentiation to avoid ambiguity. For example, while *H. pylori* is not considered a beneficial microbe in GC, its conditional immune-activating effects in specific colorectal models warrant its consideration for mechanistic exploration.

#### 6.2.1 Immunotherapy

**Figure 8:**
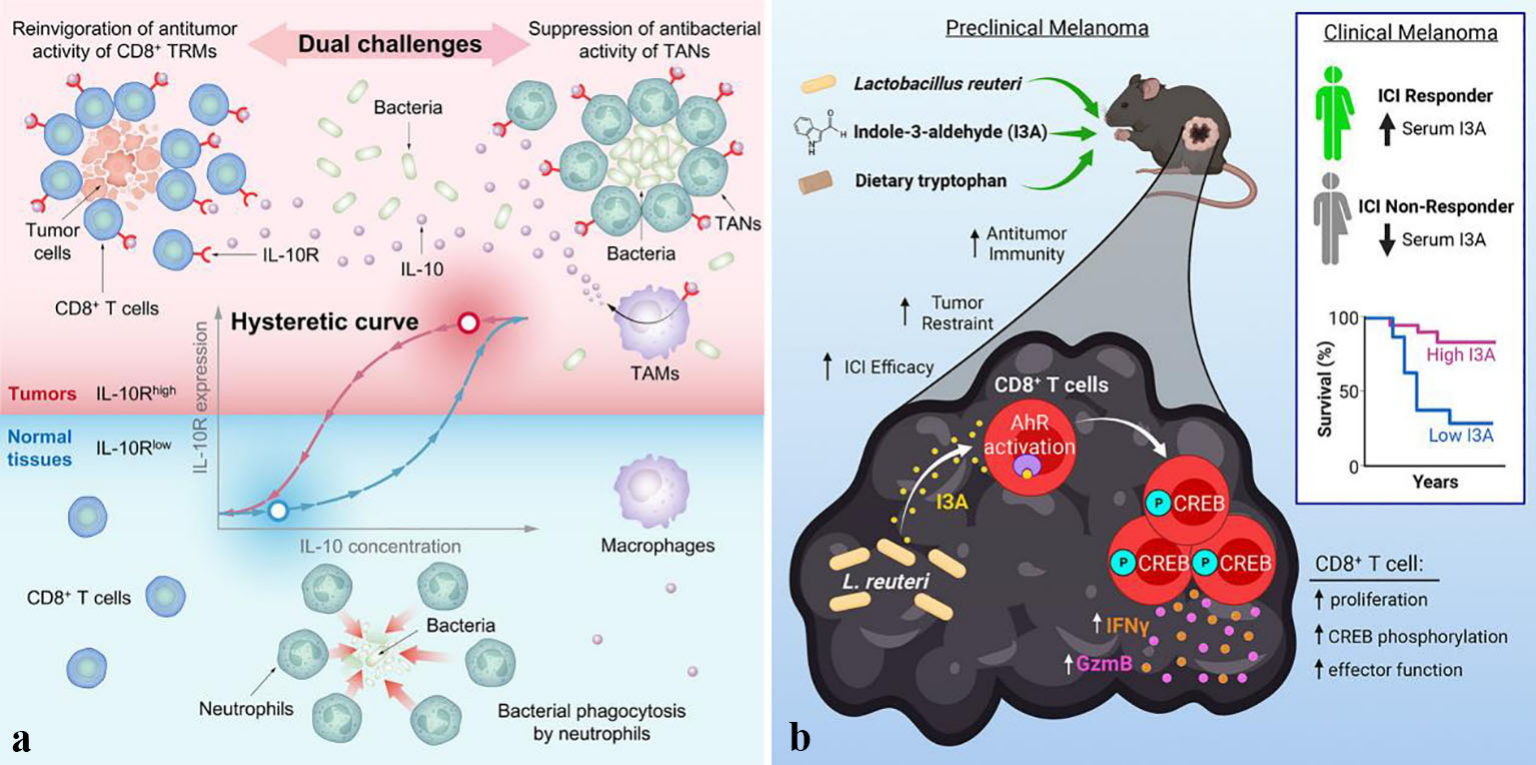
**(a)** Bacterial immunotherapy utilizing IL-10R for both phagocytosis evasion and cancer immunity revitalization and **(b)** tryptophan secreted by *Lactobacillus reuteri* boosts ICB treatment. Copyright (2023, 2025) Cell Press (Bender et al., 2023; Chang et al., 2025).

Immunotherapy represents a cornerstone of contemporary cancer treatment. Non-pathogenic intratumoral microbiota have emerged as promising agents for restoring anti-tumor immune responses, given their capacity to ameliorate the immune microenvironment and thereby enhance treatment efficacy in specific DSMTs. Several studies demonstrate that intratumoral microbiota can mediate immune activation and induce anti-tumor immunity (**Figure 8**). For example, (i) In specific experimental models (distinct from clinical settings), *H. pylori* colonization is associated with increased infiltration of CD11c+ myeloid cells, resulting in reduced CRC volume—a finding distinct from its well-documented pathogenic role in GC (Ralser et al., 2023). (ii) Inosine produced by *B. pseudolongum* enhances Th1 regulatory gene expression in CD4^+^ T cells, thereby improving anti-CRC immunotherapy (Nan and Zhong, 2025). (iii) Short-chain fatty acids and tryptophan metabolites derived from beneficial commensal bacteria activate CD8^+^ T cells and dendritic cells, concurrently diminishing myeloid-derived suppressor cells and tumor-associated macrophages within the immunosuppressive tumor microenvironment (Jagwani et al., 2025). Benign intratumoral microbiota represent promising candidates for immunotherapy and a novel avenue for clinical translation.

#### 6.2.2 Genetically engineered bacteria

**Figure 9:**
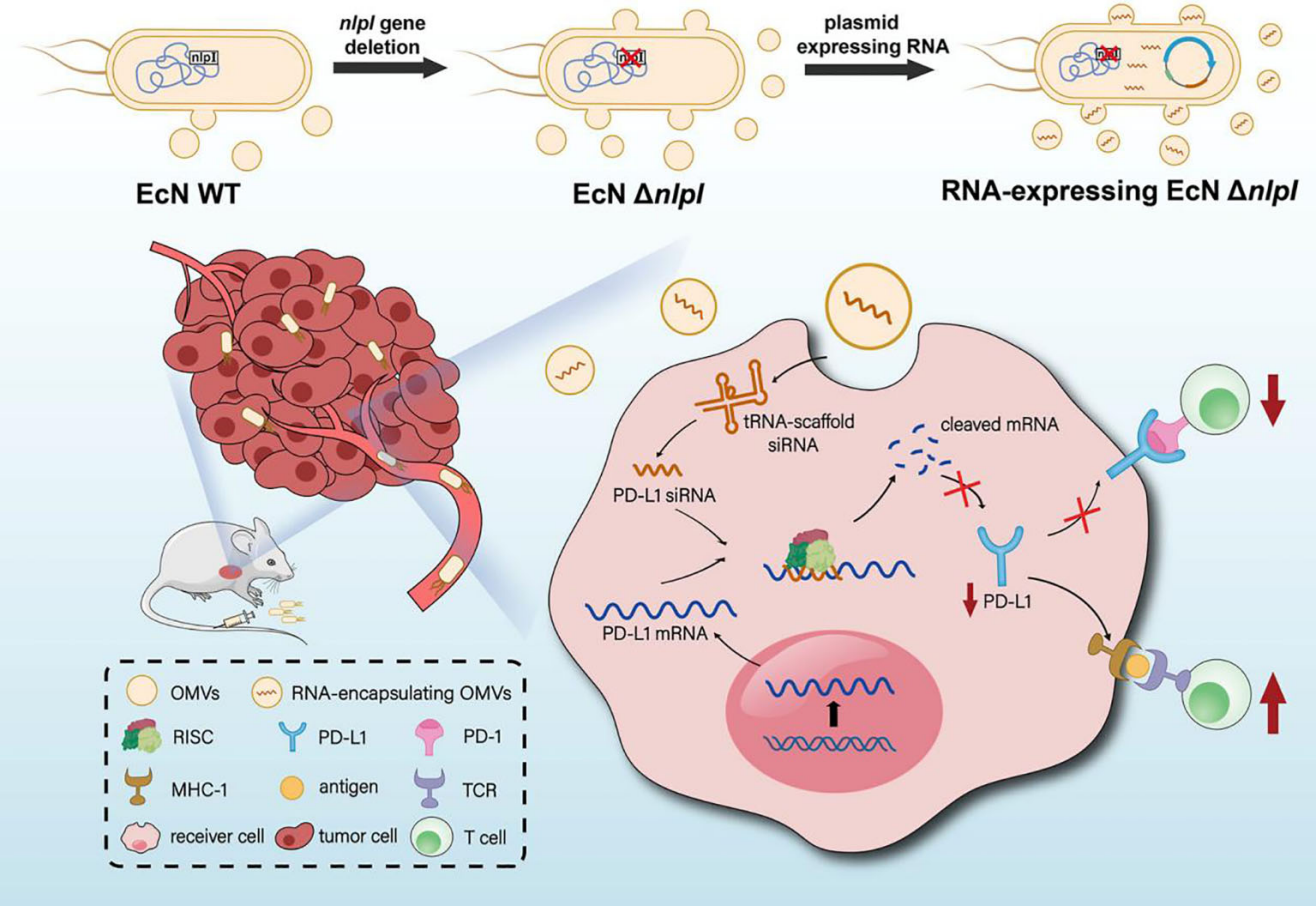
Harnessing engineered microbes as “cell-based factories” *in vivo* to facilitate intracellular self-assembly of RNA-encapsulated outer membrane vesicles in cancer treatment. Copyright (2024) American Chemical Society (Sun et al., 2024).

The intrinsic biological characteristics and safety concerns of microbiota, particularly pathogenic strains, often constrain their utility as anti-tumor agents. However, advances in genetic engineering have facilitated the development of modified microbiota exhibiting low toxicity and enhanced tumor-targeting capacity, thereby enabling their safe and effective therapeutic application (**Figure 9**) (Han et al., 2024; Sun et al., 2024). An effective strategy for enhancing anti-tumor activity involves genetically engineering bacterial vectors that secrete cytotoxic factors. For example, *Salmonella* strains expressing Fas ligand have been shown to induce potent Fas-dependent anti-tumor responses (Loeffler et al., 2008). Engineered bacteria also serve as platforms for the intratumoral delivery of cancer therapeutics. Zhu et al. developed an *L. lactis*-based *in situ* vaccine expressing a fusion protein of the co-stimulatory OX40 ligand and Fms-like tyrosine kinase 3 ligand. This vaccine activates conventional type 1 dendritic cells, cytotoxic T lymphocytes, and NK cells, and facilitates the conversion of immunologically “cold” tumors into an immunologically “hot” state, thereby enhancing anti-tumor immunity (Zhu et al., 2022). Moreover, microbiota have demonstrated favorable tolerability in cancer patients when utilized as gene delivery systems for anti-tumor therapy, and have been evaluated in numerous early-phase clinical trials. For example, Phan et al. constructed an attenuated *Salmonella typhimurium* strain containing a plasmid encoding indoleamine 2,3-dioxygenase-targeting shRNA (shIDO-ST) (Phan et al., 2020). shIDO-ST markedly enhanced CRC cell death and neutrophil infiltration.

Genetically engineered probiotics can further augment their intrinsic anti-tumor effects. For example, Savage et al. engineered *E. coli Nissle 1917* to release chemokines, including CCL20 and CXCL16 (Savage and Vincent, 2023). This not only facilitated enhanced enrichment within tumor cells but also recruited and activated adaptive (CD8+ T cells) and innate (dendritic cells) anti-tumor immune responses, thereby providing a novel strategy to enhance tumor immunotherapy.

#### 6.2.3 Intratumoral microbiota ablation technology

Targeting the microbiota with antibiotics is an established approach in tumor therapy. However, oral antibiotics can influence the systemic microenvironment by inducing intestinal microbial dysbiosis, frequently compromising the efficacy of immunotherapies. Therefore, more localized bacteriolytic strategies within tumors are required. Nanoparticle-conjugated antibiotics have demonstrated efficacy in eradicating intratumoral microbiota without disrupting systemic microbial homeostasis (Gao and Wang, 2023). Based on this, a technology using genetically engineered probiotics as carriers to deliver antibiotics for cancer treatment has emerged. This method, named the microbiota ablation technique, is currently a highly effective strategy. Current microbial ablation technologies can only target identified pathogenic microbiota supporting tumor development.

For example, amphotericin B reduces fungal colonization in PDAC cells and inhibits tumor progression. By utilizing targeted antibacterial strategies or conjugating antibiotics with nanoparticles to selectively eliminate the γ-proteobacterial population within PDAC tumors, sensitivity to gemcitabine can be restored (Jagwani et al., 2025). Genetic modification of intratumoral microbiota, combined with clinical chemotherapy, radiotherapy, and immunotherapy, is critical for treating DSMTs.

#### 6.2.4 Oncolytic microbiota

Oncolytic microbiota are natural or genetically modified microbial strains that trigger immunogenic cell death by promoting the expression of neoantigens and tumor-associated antigens, thereby eliciting adaptive and innate anti-tumor responses (Yamada et al., 2023). To date, four oncolytic viruses (e.g., T-VEC and Teserpaturev) have received clinical approval for tumor treatment. These agents have been recognized for their efficacy in precision cancer treatment. Recently, Wang et al. engineered an oncolytic vaccinia virus to express hyaluronidase and evaluated its efficacy in treating DSMTs in combination with various immunotherapeutic strategies (including CAR-T cells and monoclonal antibodies) (Wang et al., 2024). This oncolytic vaccinia virus substantially enhanced therapeutic efficacy by degrading hyaluronic acid within the TME. In CRC, tumor-derived *Cutibacterium acnes* (formerly *P. acnes*) has been recognized as an effective anti-tumor therapeutic agent (Jain and Baraniya, 2023). Similarly, Goto et al. reported the safety of intravenously administered tumor-isolated oncolytic bacteria and their inhibitory effect on CRC. While these findings suggest the potential therapeutic utility of oncolytic microbiota in tumor treatment, further investigation is warranted to investigate their efficacy and precise mechanisms of action. Moreover, it is crucial to confirm their safety profile, including the potential for systemic dissemination following off-target effects or susceptibility to inhibition by neutralizing antibodies.

## 7 TCM strategies for the prevention and treatment of DSMTs via modulation of intratumoral microbiota

### 7.1 Interactions between intratumoral microbiota and TCM

Microbiota and TCM interact in two main patterns: (i) TCM regulates microbiota makeup and metabolism, and (ii) microbiota converts TCM components. These interactions result in two types of metabolites: (i) novel metabolites produced by microbiota in response to TCM, and (ii) new TCM components formed via microbial metabolism (Xia et al., 2022).

The impacts of TCM on intratumoral microbiota are multifaceted. A typical TCM formula contains numerous compounds, such as polysaccharides, saponins, and flavonoids, which can function as prebiotics to stimulate the proliferation of specific intestinal microbial species (Wang et al., 2011). Additionally, TCM can directly inhibit microbial growth; for instance, cinnamon essential oil directly inhibits E. coliand S. aureus (Guo et al., 2024). Conversely, TCM indirectly influences the composition of intratumoral microbiota by altering the pH of the TME, thereby enhancing mucosal barrier function and inducing host immune responses (Li et al., 2018). The direct regulatory mechanisms of TCM on intratumoral microbiota remain an emerging research area, requiring further validation through furhter *in vitro*/vivo studies, and clinical investigations.

TCM can regulate microbiota metabolism by adjusting microbial composition or modulating the activity of metabolite-producing enzymes. This modulation of microbial composition and enzyme activity can result in altered levels of intrinsic metabolites and the generation of novel metabolites (Liang et al., 2023). However, microbes produce several enzymes that can transform TCM compounds. Following microbial transformation, TCM components may acquire different bioavailability and biological activity compared with their precursors (Swanson, 2015). Therefore, some TCM components previously considered inactive may exert therapeutic effects after microbial conversion (Wei et al., 2024; Zou and Wang, 2024). A comprehensive analysis of TCM–microbiota interactions is crucial for informing the rational use of TCM in intratumoral microbiota-targeted tumor treatment. Accordingly, we propose the following novel TCM-based strategies for tumor prevention and treatment.

### 7.2 New strategies of TCM for tumor prevention and treatment

Modern medicine has shifted from disease-specific to patient-specific strategies, aligning with the TCM principle of “syndrome differentiation and treatment”. We propose four strategies to integrate TCM with tumor microecology, to stimulate further reflection and discussion.

#### 7.2.1 Inhibition of “high-risk” microbiota to prevent tumor onset

TCM can prevent tumor onset by inhibiting ‘high-risk’ microbiota. Animal studies show 0.5 mg of Jianwei Xiaoshi Tablets dregs (key ingredients: luteolin and kaempferol) act via *Lactobacillus plantarum* (*L. plantarum*, HM218749) to suppress *H. pylori*, mitigate gastric mucosal damage, and restore disturbed microbiota (Meng et al., 2017). Emodin (15 mg/kg), luteolin (15 mg/kg), and paeonol (75 mg/kg) from the TCM formula Dahuang Mudan Decoction considerably inhibit *in vitro* proliferation of B. fragilis (Chen et al., 2025). Berberine (100 mg/kg) also reduces CRC incidence by suppressing intestinal *F. nucleatum* (Yu et al., 2015). However, further clinical research is needed to evaluate TCM’s effect on intratumoral microbiota.

#### 7.2.2 Promotion of beneficial bacteria growth to improve the immune TME

TCM active ingredients can enhance the body’s immune response by modulating intestinal and intratumoral microbiota, thereby promoting anti-tumor activity. Dioscin (20 mg/kg) is converted into diosgenin by intestinal microbiota. This compound exerts anti-tumor effects by optimizing gut microbial composition and enhancing T cell responses (Dong et al., 2018). The combination of Gegen Qinlian Decoction (300 mg/kg) and PD-1 antibodies effectively inhibits CRC progression (Lv et al., 2019). Preclinical mechanistic studies have demonstrated that this combination treatment significantly increases intestinal microbiota diversity, reduces PD-1 levels, and elevates CD8+ T cell counts, thereby restoring T cell function. TCM formulations commonly employed for their spleen-invigorating properties, such as Codonopsis pilosula, Atractylodes macrocephala, and Poria cocos, have been shown to increase probiotic populations, particularly Lactobacillus and Bifidobacterium (Sivan et al., 2015). These formulations can enhance the efficacy of CRC immunotherapy by optimizing the local TME. Given the diverse and complex active ingredients present in TCM compounds, large-scale clinical trials are warranted to elucidate their role in regulating tumor immunity via microbiota modulation.

#### 7.2.3 Regulation of microbiota to enhance chemotherapeutic efficacy

TCM emphasizes that “treating diseases should address the root cause, which lies in the balance of yin and yang”. This concept reflects the body’s overall yin–yang balance, including the dynamic balance of the microbiota system. When studying the tumor drug-resistant microenvironment, attention should not be limited to local effects; instead, changes in the microecology of the intestine should be evaluated simultaneously. Clinical studies show that PC tumor tissues contain *γ-Proteobacteria*. The *γ-Proteobacteria* CDD gene mediates cytidine deamination metabolism, a primary mechanism of chemoresistance in PC (Jagwani et al., 2025). Significant differences are observed in the intestinal microbiota between healthy individuals and patients, with *γ-Proteobacteria* increasing by over 1000-fold and probiotic genera such as *Bifidobacterium* being notably reduced (Pushalkar et al., 2018). Consequently, we propose that *γ-Proteobacteria* within intestinal and pancreatic tumor tissues contribute to chemoresistance in PC. Inhibiting the growth of *γ-Proteobacteria* in PC patients via antibacterial TCM may represent a promising strategy to improvegemcitabine efficacy.

#### 7.2.4 Modification of microbiota structure to improve anticancer TCM utilization

**Figure 10:**
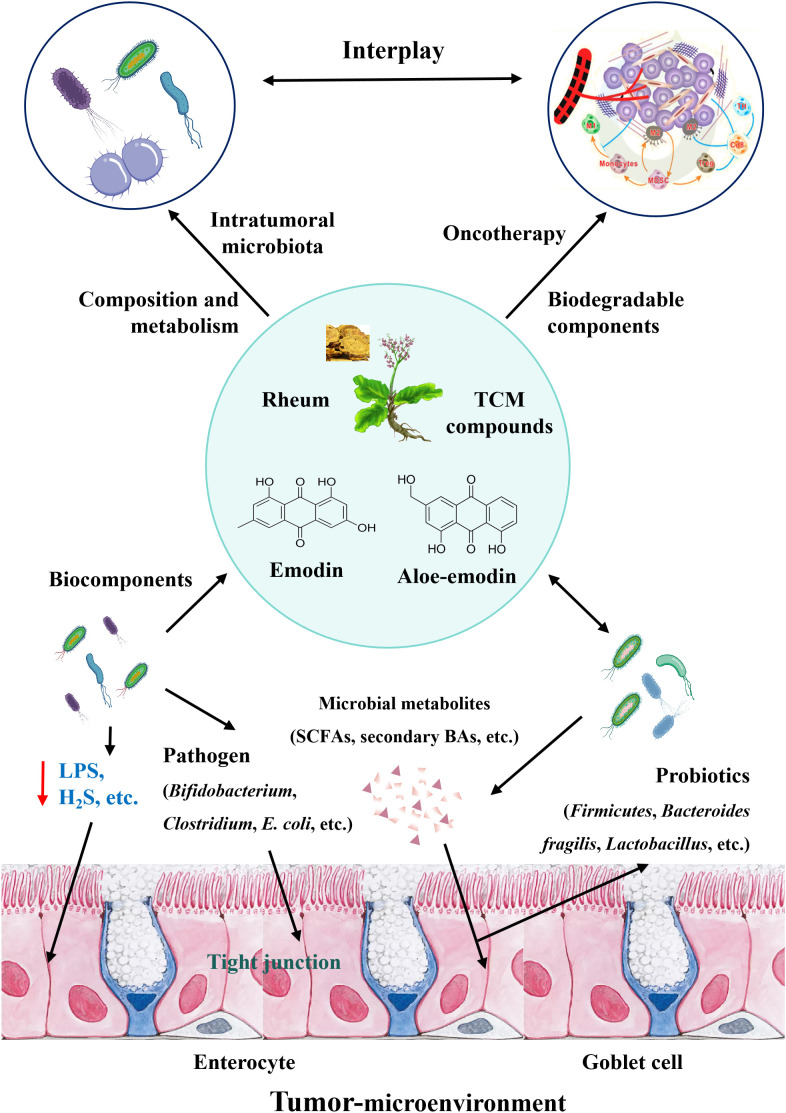
Relationship between TCM biocompounds and intratumoral microbiota. (1) TCM biocompounds can regulate the microbiota structure, and vice versa. (2) Pathogens and their metabolites can damage the intestinal barrier, while probiotics and their secreta can restore the intestinal barrier. (3) Cell barrier restoration is also beneficial for maintaining the stability of the microbial community.

The compatibility of TCM reflects its holistic principles, aiming to enhance efficacy while reducing toxicity. On one hand, adjuvant herbs (“zuo” and “shi” in TCM formulations) can regulate microbiota composition, correct dysbiosis, and facilitate probiotic proliferation. On the other hand, probiotics can transform the monarch and minister herbs (“jun” and “chen” in TCM formulations), leading to metabolites that are more readily absorbed into the bloodstream. This synergistic interaction can increase the bioavailability of anticancer TCM and reduce its toxicity. Specifically, microbiota can alter the efficacy and bioavailability of TCM through enzymatic metabolism, such as the breakdown of polysaccharides in TCM (**Figure 10**). The human body’s limited repertoire of digestive enzymes precludes the direct utilization of polysaccharides, particularly those found in TCM. However, the gut microbiota possess diverse enzymatic capabilities, enabling the degradation of these polysaccharides into metabolites that can exert therapeutic effects (Feng et al., 2018). Microbiota influence drug toxicity through enzymatic metabolism, as demonstrated with arsenic trioxide (As2O3) in leukemia treatment. Through direct metabolism, microbiota enhance arsenic’s toxicity and increase its bioavailability (Watanabe and Hirano, 2013). This strategy can be adapted to prevent and treat DSMTs using TCM.

Preclinical evidence (animal and *in vitro* studies) suggests TCM may modulate intratumoral and gut microbiota, suppressing tumor progression, enhancing immunity, and improving therapy responsiveness. Clinical evidence remains limited, requiring cautious interpretation of therapeutic efficacy. Further trials are needed to validate TCM-microbiota strategies for digestive system cancers.

## 8 Prospects and challenges

Intratumoral microbiota, as an emerging component of the TME, present novel opportunities for cancer treatment. Specifically, *F. nucleatum* has been shown to induce CRC chemoresistance, and its targeted elimination significantly enhances 5-fluorouracil efficacy. Furthermore, engineered bacterial therapies, such as attenuated *Salmonella* VNP20009 delivering IL-2, activate anti-tumor immunity and suppress metastatic progression. Microbial signatures also hold promise as biomarkers: intratumoral bacterial peptides facilitate non-invasive diagnosis and function as T cell-recognized neoantigens, while blood-based microbial analysis supports early cancer screening. Mechanistically, their metabolites exert bidirectional effects. For instance, *Lactobacillus*-derived short-chain fatty acids enhance PD-1 inhibitor responses, with some studies reporting a potential increase in immunotherapy response rates by up to 40%. Conversely, *F. nucleatum*-produced butyrate accelerates CRC progression via epigenetics mechanisms (Kong et al., 2022).

Translating these advances into clinical practice, however, faces significant challenges. From a technical perspective, the low biomass of intratumoral microbiota complicates detection, necessitating advanced techniques such as single-cell imaging and 3D quantification. Furthermore, host and environmental DNA contamination demands the development of standardized decontamination protocols. Mechanistically, the ambiguity of cause-effect relationships (e.g., the conflicting pro-oncogenic and PD-1-sensitizing roles of *F. nucleatum* in CRC) and high inter-patient heterogeneity hinder the development of universal therapies (Wang et al., 2024). Clinically, therapeutic efficacy demonstrates variability based on tumor type and immune status, while antibiotic-driven dysbiosis presents inherent safety risks. Additionally, the limitations of preclinical models in accurately mimicking human TMEs further delay the development of broad-spectrum therapies.

To bridge these gaps, encompassing both TCM and modern oncology, future research will concentrate on five interconnected directions. First, technological innovations, such as microfluidic TME-simulating culture systems, spatial multi-omics imaging, and AI-powered decontamination algorithms, will be instrumental in enhancing detection and localization. Second, in-depth mechanistic research, including live bacteria tracing and AI-driven multi-omics analysis of prospective cohorts, will be crucial for elucidating microbial migration and the interactions between TCM compounds and metabolites. Third, strategies focused on maintaining microbial balance, by distinguishing pro-tumor from anti-tumor taxa and combining targeted elimination or beneficial strain supplementation with chemo/immunotherapy, will be pivotal in ensuring safe and personalized interventions. Fourth, clinical translation will be advanced through the development of engineered bacterial “living drugs”, microbial biomarker panels, and non-invasive testing methodologies, all of which will require rigorous validation in clinical trials. Finally, robust interdisciplinary integration across fields such as microbiology, AI, and immunology will be essential to construct predictive therapeutic networks and systematically decode microbiota-tumor dynamics.

In summary, intratumoral microbiota constitute an integral component of the DSMTs’ microenvironment, exhibiting distinct compositional characteristics across various tumor types. These microbiota exert dual effects: detrimental taxa promote tumorigenesis through mechanisms such as DNA damage, oncogenic pathway activation, and immunosuppression, whereas beneficial taxa inhibit tumor progression by enhancing anti-tumor immunity and therapeutic sensitivity. This review systematically summarizes the sources, heterogeneity, and oncogenic mechanisms of intratumoral microbiota within DSMTs, integrating their diagnostic, prognostic, and therapeutic applications, with a particular emphasis on TCM-mediated regulation. Despite significant progress, substantial challenges persist. Future research endeavors should prioritize technological innovation, in-depth mechanistic exploration, and personalized intervention strategies, alongside the critical task of balancing beneficial and detrimental microbiota. Such efforts will advance microbe-centered precision oncology and ultimately improve patient prognosis for DSMTs.
