# Supplementary material for: Deciphering intratumoral microbiota in digestive system tumors: mechanisms and emerging therapeutic strategies
Source: Front Cell Infect Microbiol. 2026 Jun 10;16:1829524. doi: 10.3389/fcimb.2026.1829524 (PMC13291001; doi:10.3389/fcimb.2026.1829524)
Supplement: Supplementary file 1 [file Table1.docx]

**TABLE S1** Microbial heterogeneity and functional characteristics in different tumors.

| **Time** | **Cancer** | **Microbiota types** | **Object** | **Abundance** | **Function** | **Ref.** |
| --- | --- | --- | --- | --- | --- | --- |
| 2024 | Colorectal cancer | *Escherichia coli* | Humans | Increasing | Resulting in CRC liver metastasis | ([1](#_ENREF_1)) |
| 2021 |  | *Enterotoxigenic Bacteroides fragilis* | Mouse | Increasing | Generate reactive oxygen species and DNA damage | ([2](#_ENREF_2)) |
| 2017, 2021 |  | [*Fusobacterium nucleatum*](https://www.sciencedirect.com/topics/immunology-and-microbiology/fusobacterium-nucleatum) | Mouse | Increasing | Activates oncogenic or immunosuppressive pathways | ([3](#_ENREF_3), [4](#_ENREF_4)) |
| 2021 |  | *Colibactin-producing Escherichia coli* | Mouse | Increasing | Injure gut vascular barrier | ([5](#_ENREF_5)) |
| 2021 |  | *Firmicutes species (e.g., Clostridium, Parvimonas and Peptostreptococcus)* | Humans | Increasing | Close association with rat sarcoma viral oncogene mutation | ([6](#_ENREF_6)) |
| 2021 |  | *Campylobacter* | Humans | Increasing | Inhibite double-strand DNA break repairs | ([7](#_ENREF_7)) |
| 2018 |  | *Bifidobacterium genus* | Humans | Increasing | The amount of bifidobacteria is associated with the extent of signet ring cells | ([8](#_ENREF_8)) |
| 2021 |  | *Proteobacteria (e.g., Dechloromonas and Gallionella)* | Humans | Increasing | Positively correlated with microsatellite instability | ([9](#_ENREF_9)) |
| 2017 |  | *Peptostreptococcus anaerobius* | Mouse | Increasing | Promote cholesterol synthesis and cell proliferation | ([10](#_ENREF_10)) |
| 2015 |  | *Lactococcus, Bacteroides,*  *Prevotella, Streptococcus* | Humans | Increasing | / | ([11](#_ENREF_11)) |
| 2015 |  | *Pseudomonas,*  *Escherichia-Shigella* | Humans | Decreasing | / | ([11](#_ENREF_11)) |
| 2019 |  | *Haemophilus, Veillonella* | Humans | Increasing | / | ([12](#_ENREF_12)) |
| 2019 |  | *Saccharomycetes,*  *Pneumocystidomycetes* | Humans | Decreasing | / | ([12](#_ENREF_12)) |
| 2020 |  | *Hungatella hathewayi* | Humans and mouse | / | Upregulated DNA methyltransferases | ([13](#_ENREF_13)) |
| 2019 |  | *Akkermansia* | Mouse | / | Increased IL-17 production and B cell infiltration | ([14](#_ENREF_14)) |
| 2022 | Pancreatic cancer | [*Malassezia*](https://www.sciencedirect.com/topics/pharmacology-toxicology-and-pharmaceutical-science/malassezia) | Mouse | Increasing | Facilitate IL-33 secretion and activate ILC2 cells | ([15](#_ENREF_15)) |
| 2018 |  | *Proteobacteria, Bacteroidetes,*  *Firmicutes* | Mouse | Increasing | Altering the composition of cytokines and T cells in TME | ([16](#_ENREF_16)) |
| 2015 |  | [*Treponema denticola*](https://www.sciencedirect.com/topics/pharmacology-toxicology-and-pharmaceutical-science/treponema-denticola)*,*  [*Prevotella intermedia*](https://www.sciencedirect.com/topics/medicine-and-dentistry/prevotella-intermedia)*,*  [*Tannerella forsythia*](https://www.sciencedirect.com/topics/medicine-and-dentistry/tannerella-forsythia) | Humans | Increasing | P53 mutations | ([17](#_ENREF_17)) |
| 2019 |  | [*Aggregatibacter actinomycetemcomitans*](https://www.sciencedirect.com/topics/medicine-and-dentistry/aggregatibacter-actinomycetemcomitans) | Humans | Increasing | / | ([18](#_ENREF_18)) |
| 2018 |  | *Enterobacteriaceae, Pseudomonadaceae* | Humans | Increasing | / | ([19](#_ENREF_19)) |
| 2022 |  | *Porphyromonas gingivalis* | Mouse | Increasing | Generates a pro-inflammatory microenvironment | ([20](#_ENREF_20)) |
| 2022 |  | *Candida* | Humans | Increasing | Down-regulate genes related to cell adhesion | ([21](#_ENREF_21)) |
| 2023 |  | *Fusobacterium nucleatum* | Mouse | Increasing | Promoted PC progression through the CXCL1-CXCR2 axis | ([22](#_ENREF_22)) |
| 2018 |  | *Bifidobacterium pseudolongum* | Mouse | Increasing | Regulate TLR signaling | ([23](#_ENREF_23)) |
| 2019 |  | *Pseudoxanthomonas, Streptomyces, Saccharopolyspora,*  *Bacillus clausii* | Mouse | Increasing | Influences the host immune response | ([24](#_ENREF_24)) |
| 2024 |  | *Bacteroides, Lactobacillus,*  *Peptoniphilus* | Humans | Increasing | Suppress effector of T cells infiltration | ([25](#_ENREF_25)) |
| 2023 | Esophageal cancer | [*Fusobacterium nucleatum*](https://www.sciencedirect.com/topics/immunology-and-microbiology/fusobacterium-nucleatum) | Humans | Increasing | Alter genome-wide methylation levels | ([26](#_ENREF_26)) |
| 2024 |  | *Campylobacter, Prevotella,*  *Fusobacterium, Streptococcus* | Humans | Increasing | Promoting tumor progression by altering immune microenvironment | ([27](#_ENREF_27)) |
| 2023 | Esophageal squamous cell carcinoma | [*Fusobacterium nucleatum*](https://www.sciencedirect.com/topics/immunology-and-microbiology/fusobacterium-nucleatum) | Humans | Increasing | Suppress the proliferation and cytokine secretion of T cells | ([28](#_ENREF_28)) |
| 2023 |  | *Streptococcus* | Humans | Decreasing | Predicting poor neoadjuvant chemoimmunotherapy response | ([29](#_ENREF_29)) |
| 2023 |  | *Lactobacillus* | Humans | Increasing | Predicting long-term survival | ([30](#_ENREF_30)) |
| 2020 |  | *Porphyromonas gingivalis* | Humans | Increasing | Associate with advanced clinical stages and a poor prognosis. | ([31](#_ENREF_31)) |
| 2023 | Hepatocellular carcinoma | [*Mycoplasma*](https://www.sciencedirect.com/topics/pharmacology-toxicology-and-pharmaceutical-science/mycoplasma)*hyorhinis* | Humans | Increasing | Stimulate cancer stemness by boosting mitochondrial fission | ([32](#_ENREF_32)) |
| 2023 |  | *Enterobacteriaceae* | Humans | Increasing | Associate with higher levels of inflammation | ([33](#_ENREF_33)) |
| 2023 |  | *Neisseria* | Humans | Increasing | / | ([33](#_ENREF_33)) |
| 2023 |  | *Fusobacterium* | Humans | Increasing | Transport non-invasive bacteria into the host cells and promote the inflammatory response | ([33](#_ENREF_33)) |
| 2023 |  | *Pseudomonas* | Humans | Decreasing | / | ([33](#_ENREF_33)) |
| 2018, 2012, 2021, 2022 |  | *Hepatitis B virus* | Humans, mouse and hepatic cells | Increasing | Integrate viral genome into the chromosome; Promoted m6A modification; Recruited regulatory T cells | ([34-36](#_ENREF_34)) |
| 2018, 2019 |  | *Hepatitis C virus* | Humans, mouse and hepatic cells | Increasing | Recruited regulatory T cells | ([37](#_ENREF_37), [38](#_ENREF_38)) |
| 2022 |  | *Order Gammaproteobacteria* | humans | Increasing | / | ([39](#_ENREF_39)) |
| 2022 |  | *Streptococcaceae, Lactococcus* | Humans | Increasing | / | ([39](#_ENREF_39)) |
| 2022 |  | *Caulobacteraceae,*  *Rickettsiaceae* | Humans | Decreasing | / | ([40](#_ENREF_40)) |
| 2024 |  | *Cutibacterium* | Humans | Increasing | Related to hepatitis B virus-HCC and tumor immunity promotion | ([41](#_ENREF_41)) |
| 2023 |  | *Akkermansia muciniphila,*  *Methylobacterium* | Humans | Increasing | Associated with favorable OS and RFS | ([42](#_ENREF_42)) |
| 2023 | Intrahepatic cholangiocarcinoma | *Paraburkholderia fungorum* | Humans and mouse | Decreasing | Impact tumor growth by engaging in amino acid metabolic pathways | ([43](#_ENREF_43)) |
| 2023 |  | *Pseudomonas azotoformans* | Humans | Increasing | / | ([43](#_ENREF_43)) |
| 2023 |  | *Staphylococcus capitis* | Humans | Increasing | / | ([43](#_ENREF_43)) |
| 2024 | Gastric cancer | [*Streptococcus anginosus*](https://www.sciencedirect.com/topics/medicine-and-dentistry/streptococcus-anginosus) | Mouse | Increasing | Disrupte gastric barrier function | ([44](#_ENREF_44)) |
| 2022 |  | *Methylobacterium* | Mouse | Increasing | Decrease TGFβ expression and CD8+ TRM cells | ([45](#_ENREF_45)) |
| 2010 |  | *Helicobacter pylori* | Mouse | Increasing | Aberrantly activates oncogenic signalling pathways | ([46](#_ENREF_46)) |
| 2022 |  | *Lactobacillus, Prevotella,*  *Bacteroides* | Humans | Increasing | / | ([47](#_ENREF_47)) |
| 2014 |  | *Phylum TM7, Porphyromonas,*  *Neisseria, Streptococcus sinensis* | Humans | Decreasing | / | ([48](#_ENREF_48)) |
| 2014 |  | *Family Lachnospiraceae,*  *Lactobacillus coleohominis* | Humans | Increasing | / | ([48](#_ENREF_48)) |

**TABLE S2** Summary of Published Clinical Trials of Antitumor Bacterial and Viral Therapies for DSMTs.

| **Species** | **Product** | **Type** | **Administration** | **Cancer** | **Trial** | **Number of subjects** | **Result** | **NCT Number** |
| --- | --- | --- | --- | --- | --- | --- | --- | --- |
| *Adenovirus* | H101 | Oncolytic viral | / | Pancreatic ductal adenocarcinoma | A Phase 1b Dose-escalation, Cohort-expansion Study | 56 | No Results Posted | NCT05303090 |
| *Listeria monocytogenes* | CRS-207 | Live attenuated | Intravenous | Pancreatic cancer | Randomized, phase II trial, multicenter | 90 | Trigger mesothelin-specific CD8 T cell responses and  extend patients survival | NCT02243371 |
|  |  |  |  |  | Phase IIb trial, randomized | 303 | Provide comparable survival benefits with standard chemotherapy | NCT03006302 |
| *Multiple bacteria* | Mixed Bacterial Vaccine | Live attenuated | Subcutaneous injection or Intratumoral injection | Gastrointestinal stromal tumor, esophageal cancer | Single-arm study, multiple dose, phase 1 | 17 | Result in a substantial induction of immunoregulatory cytokines that may contribute to tumor regressions | NCT00623831 |
| *Salmonella typhi* | VXM01 | Live attenuated | Oral | Advanced pancreatic cancer | Randomized, placebocontrolled, phase I doseescalation trial | 26 | Induce vaccine specific T cell responses | NCT01486329 |
|  |  |  |  |  | Randomized, dose-escalation phase I clinical trial | 30 | Trigger vaccine specific T_eff_ cell responses and decrease tumor perfusion | NCT01486329 |
| *Salmonella typhimurium expressing IL-2* | SalpIL2 |  |  | Liver cancer, biliary cancer | Nonrandomized, open-label,dose-escalation phase I clinical trial | 22 | / | NCT01099631 |
| *Vaccina virus* | PexaVec | Oncolytic viral | Intravenous injection | Colorectal cancer | Clinical Trial | 34 | Monitoring the safety and tolerance profiles in a phase I/II of CRC | NCT03206073 |
| *Vaccinia virus PexaVec* | JX594 |  |  | Primary or metastatic hepatic carcinoma | Phase I, dose-escalation | 14 | No tumor regrowth | NCT00629759 |

**References**

**1.** Gu, J., X. Xu, X. Li, L. Yue, X. Zhu, Q. Chen, et al. (2024). Tumor-resident microbiota contributes to colorectal cancer liver metastasis by lactylation and immune modulation. *Oncogene*. 43, 2389-2404. doi:10.1038/s41388-024-03080-7.

**2.** Qu, R., Y. Zhang, Y. Ma, X. Zhou, L. Sun, C. Jiang, et al. (2023). Role of the Gut Microbiota and Its Metabolites in Tumorigenesis or Development of Colorectal Cancer. *Adv Sci (Weinh)*. 10, e2205563. doi:10.1002/advs.202205563.

**3.** Kong, C., X. Yan, Y. Zhu, H. Zhu, Y. Luo, P. Liu, et al. (2021). Fusobacterium Nucleatum Promotes the Development of Colorectal Cancer by Activating a Cytochrome P450/Epoxyoctadecenoic Acid Axis via TLR4/Keap1/NRF2 Signaling. *Cancer Res*. 81, 4485-4498. doi:10.1158/0008-5472.can-21-0453.

**4.** Yu, T., F. Guo, Y. Yu, T. Sun, D. Ma, J. Han, et al. (2017). Fusobacterium nucleatum Promotes Chemoresistance to Colorectal Cancer by Modulating Autophagy. *Cell*. 170, 548-563. doi:10.1016/j.cell.2017.07.008.

**5.** Bertocchi, A., S. Carloni, P. S. Ravenda, G. Bertalot, I. Spadoni, A. Lo Cascio, et al. (2021). Gut vascular barrier impairment leads to intestinal bacteria dissemination and colorectal cancer metastasis to liver. *Cancer Cell*. 39, 708-724. doi:10.1016/j.ccell.2021.03.004.

**6.** Zhang, J., P. Wang, J. Wang, X. Wei and M. Wang. (2024). Unveiling intratumoral microbiota: An emerging force for colorectal cancer diagnosis and therapy. *Pharmacol Res*. 203, 107185. doi:10.1016/j.phrs.2024.107185.

**7.** Miyazono, K. I., D. Wang, T. Ito and M. Tanokura. (2021). Crystal structure and DNA cleavage mechanism of the restriction DNA glycosylase R.CcoLI from Campylobacter coli. *Sci Rep*. 11, 859. doi:10.1038/s41598-020-79537-y.

**8.** Kosumi, K., T. Hamada, H. Koh, J. Borowsky, S. Bullman, T. S. Twombly, et al. (2018). The Amount of Bifidobacterium Genus in Colorectal Carcinoma Tissue in Relation to Tumor Characteristics and Clinical Outcome. *Am J Pathol*. 188, 2839-2852. doi:10.1016/j.ajpath.2018.08.015.

**9.** Giammanco, A., R. Anzalone and N. Serra. (2023). Helicobacter pylori and Epstein-Barr Virus Co-Infection in Gastric Disease: What Is the Correlation with p53 Mutation, Genes Methylation and Microsatellite Instability in a Cohort of Sicilian Population? *Int J Mol Sci*. 24, 8104. doi:10.3390/ijms24098104.

**10.** Tsoi, H., E. S. H. Chu, X. Zhang, J. Sheng, G. Nakatsu, S. C. Ng, et al. (2017). Peptostreptococcus anaerobius Induces Intracellular Cholesterol Biosynthesis in Colon Cells to Induce Proliferation and Causes Dysplasia in Mice. *Gastroenterology*. 152, 1419-1433. doi:10.1053/j.gastro.2017.01.009.

**11.** Oggesen, B. T., M. L. S. Hamberg and J. Rosenberg. (2023). Practical management algorithms for late complications after colorectal and anal cancer—Basic treatment of late complications. *Medicine Advances*. 1, 260-269. doi:https://doi.org/10.1002/med4.32.

**12.** Coker, O. O., G. Nakatsu, R. Z. Dai, W. K. K. Wu and S. H. Wong. (2019). Enteric fungal microbiota dysbiosis and ecological alterations in colorectal cancer. *Gut*. 68, 654-662. doi:10.1136/gutjnl-2018-317178.

**13.** Xia, X., W. K. K. Wu, S. H. Wong, D. Liu, T. N. Y. Kwong, G. Nakatsu, et al. (2020). Bacteria pathogens drive host colonic epithelial cell promoter hypermethylation of tumor suppressor genes in colorectal cancer. *Microbiome*. 8, 108. doi:10.1186/s40168-020-00847-4.

**14.** Triner, D., S. N. Devenport, S. K. Ramakrishnan, X. Ma, R. A. Frieler, J. K. Greenson, et al. (2019). Neutrophils Restrict Tumor-Associated Microbiota to Reduce Growth and Invasion of Colon Tumors in Mice. *Gastroenterology*. 156, 1467-1482. doi:10.1053/j.gastro.2018.12.003.

**15.** Alam, A., E. Levanduski, P. Denz, H. S. Villavicencio, M. Bhatta, L. Alhorebi, et al. (2022). Fungal mycobiome drives IL-33 secretion and type 2 immunity in pancreatic cancer. *Cancer Cell*. 40, 153-167.e11. doi:10.1016/j.ccell.2022.01.003.

**16.** Sethi, V., S. Kurtom, M. Tarique, S. Lavania, Z. Malchiodi, L. Hellmund, et al. (2018). Gut Microbiota Promotes Tumor Growth in Mice by Modulating Immune Response. *Gastroenterology*. 155, 33-37. doi:10.1053/j.gastro.2018.04.001.

**17.** Öğrendik, M. (2015). Oral bacteria in pancreatic cancer: mutagenesis of the p53 tumour suppressor gene. *Int J Clin Exp Pathol*. 8, 11835-6.

**18.** Gaiser, R. A., A. Halimi, H. Alkharaan, L. Lu, H. Davanian, K. Healy, et al. (2019). Enrichment of oral microbiota in early cystic precursors to invasive pancreatic cancer. *Gut*. 68, 2186-2194. doi:10.1136/gutjnl-2018-317458.

**19.** Geller, L. T. and M. Barzily-Rokni. (2017). Potential role of intratumor bacteria in mediating tumor resistance to the chemotherapeutic drug gemcitabine. *Science*. 357, 1156-1160. doi:10.1126/science.aah5043.

**20.** Tan, Q., X. Ma, B. Yang, Y. Liu, Y. Xie, X. Wang, et al. (2022). Periodontitis pathogen Porphyromonas gingivalis promotes pancreatic tumorigenesis via neutrophil elastase from tumor-associated neutrophils. *Gut Microbes*. 14, 2073785. doi:10.1080/19490976.2022.2073785.

**21.** Dohlman, A. B., J. Klug, M. Mesko, I. H. Gao, S. M. Lipkin, X. Shen, et al. (2022). A pan-cancer mycobiome analysis reveals fungal involvement in gastrointestinal and lung tumors. *Cell*. 185, 3807-3822. doi:10.1016/j.cell.2022.09.015.

**22.** Hayashi, M. and N. Ikenaga. (2023). Intratumor Fusobacterium nucleatum promotes the progression of pancreatic cancer via the CXCL1-CXCR2 axis. *Cancer Sci*. 114, 3666-3678. doi:10.1111/cas.15901.

**23.** Pushalkar, S., M. Hundeyin, D. Daley, C. P. Zambirinis, E. Kurz, A. Mishra, et al. (2018). The Pancreatic Cancer Microbiome Promotes Oncogenesis by Induction of Innate and Adaptive Immune Suppression. *Cancer Discov*. 8, 403-416. doi:10.1158/2159-8290.cd-17-1134.

**24.** Riquelme, E., Y. Zhang, L. Zhang, M. Montiel, M. Zoltan, W. Dong, et al. (2019). Tumor Microbiome Diversity and Composition Influence Pancreatic Cancer Outcomes. *Cell*. 178, 795-806. doi:10.1016/j.cell.2019.07.008.

**25.** Abe, S. and A. Masuda. (2024). Impact of intratumoral microbiome on tumor immunity and prognosis in human pancreatic ductal adenocarcinoma. *J Gastroenterol*. 59, 250-262. doi:10.1007/s00535-023-02069-5.

**26.** Baba, Y., Y. Hara, T. Toihata, K. Kosumi, M. Iwatsuki, S. Iwagami, et al. (2023). Relationship between gut microbiome Fusobacterium nucleatum and LINE-1 methylation level in esophageal cancer. *Esophagus*. 20, 704-712. doi:10.1007/s10388-023-01009-9.

**27.** Greathouse, K. L., J. K. Stone, A. J. Vargas, A. Choudhury, R. N. Padgett, J. R. White, et al. (2024). Co-enrichment of cancer-associated bacterial taxa is correlated with immune cell infiltrates in esophageal tumor tissue. *Sci Rep*. 14, 2574. doi:10.1038/s41598-023-48862-3.

**28.** Li, Y. and S. Xing. (2023). Intracellular Fusobacterium nucleatum infection attenuates antitumor immunity in esophageal squamous cell carcinoma. *Nat Commun*. 14, 5788. doi:10.1038/s41467-023-40987-3.

**29.** Wu, H. and X. Leng. (2023). Intratumoral Microbiota Composition Regulates Chemoimmunotherapy Response in Esophageal Squamous Cell Carcinoma. *Cancer Res*. 83, 3131-3144. doi:10.1158/0008-5472.can-22-2593.

**30.** Zhang, S., S. Zhang, X. Ma, J. Zhan, C. Pan, H. Zhang, et al. (2023). Intratumoral microbiome impacts immune infiltrates in tumor microenvironment and predicts prognosis in esophageal squamous cell carcinoma patients. *Front Cell Infect Microbiol*. 13, 1165790. doi:10.3389/fcimb.2023.1165790.

**31.** Chen, M. F., M. S. Lu, C. C. Hsieh and W. C. Chen. (2021). Porphyromonas gingivalis promotes tumor progression in esophageal squamous cell carcinoma. *Cell Oncol (Dordr)*. 44, 373-384. doi:10.1007/s13402-020-00573-x.

**32.** Qiao, K., J. Han, H. Zhang, Y. Li, X. Hou, Y. Jia, et al. (2023). Intratumor Mycoplasma promotes the initiation and progression of hepatocellular carcinoma. *Cell Rep*. 42, 113563. doi:10.1016/j.celrep.2023.113563.

**33.** He, Y., Q. Zhang, X. Yu, S. Zhang and W. Guo. (2023). Overview of microbial profiles in human hepatocellular carcinoma and adjacent nontumor tissues. *J Transl Med*. 21, 68. doi:10.1186/s12967-023-03938-6.

**34.** Jiang, Z., S. Jhunjhunwala, J. Liu, P. M. Haverty, M. I. Kennemer, Y. Guan, et al. (2012). The effects of hepatitis B virus integration into the genomes of hepatocellular carcinoma patients. *Genome Res*. 22, 593-601. doi:10.1101/gr.133926.111.

**35.** Kostyusheva, A., S. Brezgin, D. Glebe, D. Kostyushev and V. Chulanov. (2021). Host-cell interactions in HBV infection and pathogenesis: the emerging role of m6A modification. *Emerg Microbes Infect*. 10, 2264-2275. doi:10.1080/22221751.2021.2006580.

**36.** Gao, Y., M. You, J. Fu, M. Tian, X. Zhong, C. Du, et al. (2022). Intratumoral stem-like CCR4+ regulatory T cells orchestrate the immunosuppressive microenvironment in HCC associated with hepatitis B. *J Hepatol*. 76, 148-159. doi:10.1016/j.jhep.2021.08.029.

**37.** Maucort-Boulch, D. and C. de Martel. (2018). Fraction and incidence of liver cancer attributable to hepatitis B and C viruses worldwide. *Int J Cancer*. 142, 2471-2477. doi:10.1002/ijc.31280.

**38.** Ouaguia, L. and O. Moralès. (2019). Hepatitis C Virus Improves Human Tregs Suppressive Function and Promotes Their Recruitment to the Liver. *Cells*. 8. doi:10.3390/cells8101296.

**39.** Huang, J. H., J. Wang, X. Q. Chai, Z. C. Li, Y. H. Jiang, J. Li, et al. (2022). The Intratumoral Bacterial Metataxonomic Signature of Hepatocellular Carcinoma. *Microbiol Spectr*. 10, e0098322. doi:10.1128/spectrum.00983-22.

**40.** Qu, D., Y. Wang, Q. Xia, J. Chang, X. Jiang and H. Zhang. (2022). Intratumoral Microbiome of Human Primary Liver Cancer. *Hepatol Commun*. 6, 1741-1752. doi:10.1002/hep4.1908.

**41.** Liu, Y., E. S. Kim and H. Guo. (2024). Hepatitis B virus-related hepatocellular carcinoma exhibits distinct intratumoral microbiota and immune microenvironment signatures. *J Med Virol*. 96, e29485. doi:10.1002/jmv.29485.

**42.** Sun, L., X. Ke, A. Guan, B. Jin, J. Qu, Y. Wang, et al. (2023). Intratumoural microbiome can predict the prognosis of hepatocellular carcinoma after surgery. *Clin Transl Med*. 13, e1331. doi:10.1002/ctm2.1331.

**43.** Chai, X., J. Wang, H. Li, C. Gao, S. Li, C. Wei, et al. (2023). Intratumor microbiome features reveal antitumor potentials of intrahepatic cholangiocarcinoma. *Gut Microbes*. 15, 2156255. doi:10.1080/19490976.2022.2156255.

**44.** Fu, K., A. H. K. Cheung, C. C. Wong, W. Liu, Y. Zhou, F. Wang, et al. (2024). Streptococcus anginosus promotes gastric inflammation, atrophy, and tumorigenesis in mice. *Cell*. 187, 882-896. doi:10.1016/j.cell.2024.01.004.

**45.** Peng, R. and S. Liu. (2022). Gastric Microbiome Alterations Are Associated with Decreased CD8+ Tissue-Resident Memory T Cells in the Tumor Microenvironment of Gastric Cancer. *Cancer Immunol Res*. 10, 1224-1240. doi:10.1158/2326-6066.cir-22-0107.

**46.** Elinav, E., R. Nowarski, C. A. Thaiss, B. Hu, C. Jin and R. A. Flavell. (2013). Inflammation-induced cancer: crosstalk between tumours, immune cells and microorganisms. *Nat Rev Cancer*. 13, 759-71. doi:10.1038/nrc3611.

**47.** Abate, M., E. Vos, M. Gonen, Y. Y. Janjigian, M. Schattner, M. Laszkowska, et al. (2022). A Novel Microbiome Signature in Gastric Cancer: A Two Independent Cohort Retrospective Analysis. *Ann Surg*. 276, 605-615. doi:10.1097/sla.0000000000005587.

**48.** Aviles-Jimenez, F., F. Vazquez-Jimenez, R. Medrano-Guzman, A. Mantilla and J. Torres. (2014). Stomach microbiota composition varies between patients with non-atrophic gastritis and patients with intestinal type of gastric cancer. *Sci Rep*. 4, 4202. doi:10.1038/srep04202.
